# The Effects of 6 Common Antidiabetic Drugs on Anti-PD1 Immune Checkpoint Inhibitor in Tumor Treatment

**DOI:** 10.1155/2022/2651790

**Published:** 2022-08-18

**Authors:** Ze-Tao Zhan, Lu Liu, Ming-Zhen Cheng, Yi Gao, Wei-Jie Zhou

**Affiliations:** ^1^State Key Laboratory of Organ Failure Research, Department of General Surgery & Guangdong Provincial Key Laboratory of Precision Medicine for Gastrointestinal Tumor, Department of Pathology, Nanfang Hospital, School of Basic Medical Sciences, Southern Medical University, Guangzhou, China; ^2^General Surgery Center, Department of Hepatobiliary Surgery II, Guangdong Provincial, Research Center for Artificial Organ and Tissue Engineering, Guangzhou Clinical Research and Transformation Center for Artificial Liver, Institute of Regenerative Medicine, Zhujiang Hospital, Southern Medical University, Guangzhou, China; ^3^Microbiome Medicine Center, Zhujiang Hospital, Southern Medical University, Guangzhou, China; ^4^Bioland Laboratory (Guangzhou Regenerative Medicine and Health Guangdong Laboratory), Guangzhou, China

## Abstract

Diabetes and cancer are common diseases and are frequently diagnosed in the same individual. These patients need to take antidiabetic drugs while receiving antitumor drugs therapy. Recently, immunotherapy offers significant advances for cancer treatment. However, it is unclear whether antidiabetic drugs affect immunotherapy. Here, by employing syngeneic mouse colon cancer model and melanoma model, we studied the effects of 6 common antidiabetic drugs on anti-PD1 immune checkpoint inhibitor in tumor treatment, including acarbose, sitagliptin, metformin, glimepiride, pioglitazone, and insulin. We found that acarbose and sitagliptin enhanced the tumor inhibition of anti-PD1, and metformin had no effect on the tumor inhibition of anti-PD1, whereas glimepiride, pioglitazone, and insulin weakened the tumor inhibition of anti-PD1. Our study suggests that cancer patients receiving anti-PD1 antibody therapy need serious consideration when choosing antidiabetic drugs. In particular, acarbose significantly inhibited tumor growth and further enhanced the therapeutic effect of anti-PD1, which can be widely used in tumor therapy. Based on this study, further clinical trials are expected.

## 1. Introduction

Diabetes mellitus and tumor are common diseases, and their codiagnosis in the same individual is frequent. More than 400 million people are diagnosed with diabetes worldwide [1, 2]. Increasing epidemiological studies show that diabetes is positively correlated with the risk of most common malignant tumors, including colon, liver, breast, endometrium, bladder, lung, and pancreas cancer [3–7]. Only prostatic cancer occurs less frequently in patients with diabetes [8, 9]. In 2020, there were an estimated 19.3 million new cancer cases and 10 million cancer deaths worldwide [10]. According to this ratio, it is estimated that at least 1.1 million new cancer cases would be codiagnosed with diabetes mellitus in 2020. Furthermore, it is suggested that the mortality rate of patients with diabetes increased, compared with those with normal glycemic of all cancer types, especially in patients with endometrium, breast, and colorectal cancer [11, 12]. Therefore, controlling the diabetes condition of cancer patients is necessary.

Different antidiabetic drugs have different effects on risk of cancer. Metformin has been reported to decrease cancer risk or cancer mortality [13], whereas insulin and sulfonylureas might be associated with increased cancer risk [14, 15]. The results for thiazolidinediones are controversial, which may increase [16], decrease [17], or have a neutral effect [18] on the risk of cancer or cancer progression. Recently, immunotherapy has become a powerful clinical strategy for cancer treatment. Checkpoint blockade are the most thoroughly investigated class of immunotherapy so far. PD-1/PD-L1 blockade is one of the most common checkpoint inhibition strategies. To escape recognition and elimination by T cells, tumor cells express PD-L1, which binds to PD-1 on T cells to inactivate these cells. Therefore, blocking this interaction with monoclonal antibodies (mAbs) targeting either PD-1 or PD-L1 leads to T cell-mediated tumor cell death [19–21]. The clinical impact of PD-1/PD-L1 checkpoint blockade strategy has been growing over the past few years. To date, five PD-1 or PD-L1 inhibitors have been approved to treat various cancers with improved overall survival compared with traditional chemotherapies [22]. However, it is unclear whether antidiabetic drugs have synergistic or antagonistic effects on PD/PDL1 inhibitors. Understanding this question will help cancer patients codiagnosed with diabetes to choose the right antidiabetic drugs when using PD/PDL1 inhibitors to treat tumors.

In this study, using syngeneic mouse colon cancer and melanoma models, we examined the effects of 6 common antidiabetic drugs on tumor inhibition of anti-PD1, including acarbose, sitagliptin, metformin, glimepiride, pioglitazone, and insulin. Our results showed that acarbose and sitagliptin enhanced the effect of anti-PD1 on tumor inhibition, metformin had no effect on the effect of anti-PD1 on tumor inhibition, whereas glimepiride, pioglitazone, and insulin weakened the effect of anti-PD1 on tumor inhibition.

## 2. Results

### 2.1. Acarbose Inhibits Tumor Growth and Enhances Tumor Immune Responses to Anti-PD1

**Figure 1:**
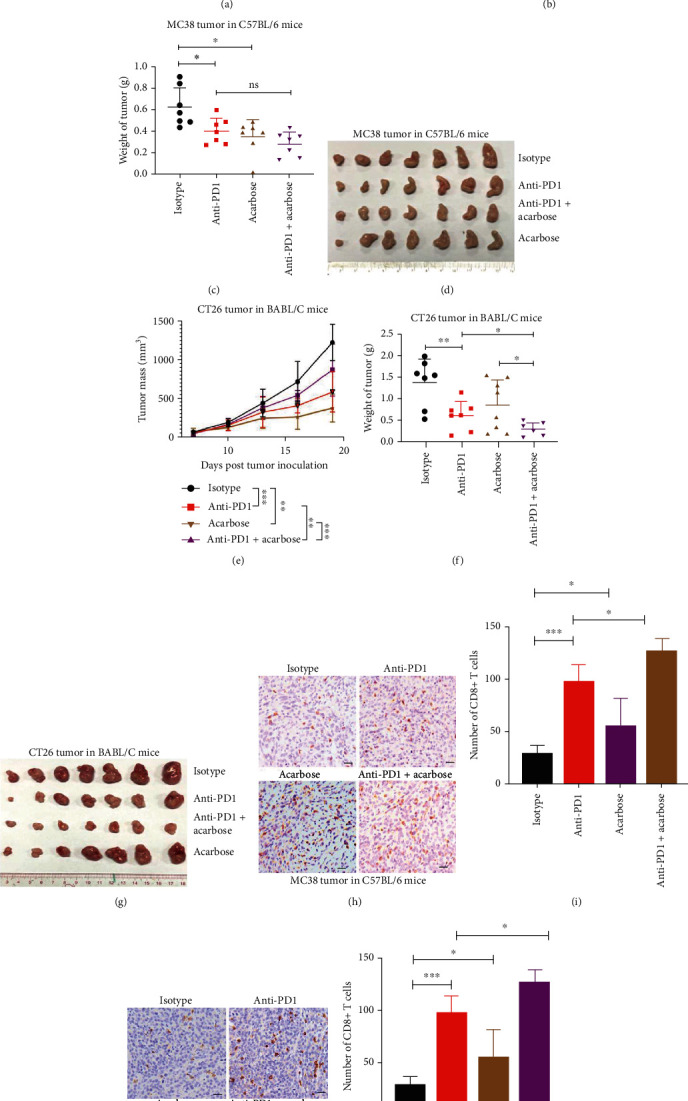
Acarbose inhibits colon tumor growth and enhances tumor immune responses to anti-PD1. (a) Schematic overview of experimental design combined for (b)–(k). (b–d) Tumor growth (b) and weight (c) from MC38 tumor-bearing C57BL/6 mice were shown (*n* = 7). (e–g) Tumor growth (e) and weight (f) from CT26 tumor-bearing on BABL/C mice were shown (*n* = 7). (h and i) Sections from MC38 subcutaneous tumors were stained with anti-CD8 antibody by immunohistochemistry (h), and CD8^+^ T cells infiltration were analyzed in (i). (j and k) Sections from CT26 subcutaneous tumors were stained with anti-CD8 antibody by immunohistochemistry (j), and CD8^+^ T cells infiltration were analyzed in (k). Scale bars: 100 *μ*m. Data were analyzed by two-way ANOVA (b and e) and *t*-test (c, f, i, and k). ns: no significance; ^∗^*p* < 0.05, ^∗∗^*p* < 0.01, and ^∗∗∗^*p* < 0.001. Error bars denote for the s.e.m.

Acarbose is an *α*-glucosidase inhibitor, which arrests *α*-glucosidase activity of intestinal wall cells by competing with oligosaccharides to delay the process of carbohydrate degradation and effectively slow the absorption of glucose by pancreatic tissues [23]. In experiments with a syngeneic mouse colon cancer model (Figure 1(a)), acarbose monotherapy and mouse anti-PD1 immune checkpoint inhibitor monotherapy significantly inhibited the growth of subcutaneously grafted MC38 cells in WT C57BL/6 mice, respectively (Figures 1(b)–1(d)). Furthermore, the combination of acarbose and anti-PD1 showed a more significant effect on tumor growth inhibition (Figures 1(b)–1(d)). Similar results were observed in experiments using mouse colon cancer CT26 cells. Acarbose monotherapy and anti-PD1 monotherapy significantly inhibited the growth of subcutaneously grafted CT26 cells in WT Balb/c mice, respectively (Figures 1(e)–1(g)), and the combination of acarbose and anti-PD1 showed a more significant effect on tumor growth inhibition (Figures 1(e)–1(g)). Immunohistological examination of the MC38 and CT26 tumors showed that acarbose and anti-PD1 significantly increased the number of tumoral infiltrated CD8^+^ T cells, respectively (Figures 1(h)–1(k)), and the combination of acarbose and anti-PD1 induced more tumoral infiltrated CD8^+^ T cells in tumors (Figures 1(h)–1(k)).

In experiments with a syngeneic mouse melanoma cancer model (Figure S1A), acarbose significantly inhibited the growth of subcutaneously grafted B16 cells in WT C57BL/6 mice, while anti-PD1 had no effect on the B16 tumor growth (Figure S1B-D). Surprisingly, the combination of acarbose and anti-PD1 showed an enhanced effect on tumor growth inhibition (Figure S1B–D). Immunohistological examination of the B16 tumors showed that acarbose and anti-PD1 significantly increased the number of tumoral infiltrated CD8^+^ T cells, respectively (Figure S1E-H), and the combination of acarbose and anti-PD1 induced more tumoral infiltrated CD8^+^ T cells in tumors (Figure S1E-H).

### 2.2. Sitagliptin Shows Different Roles in Different Tumor Models

**Figure 2:**
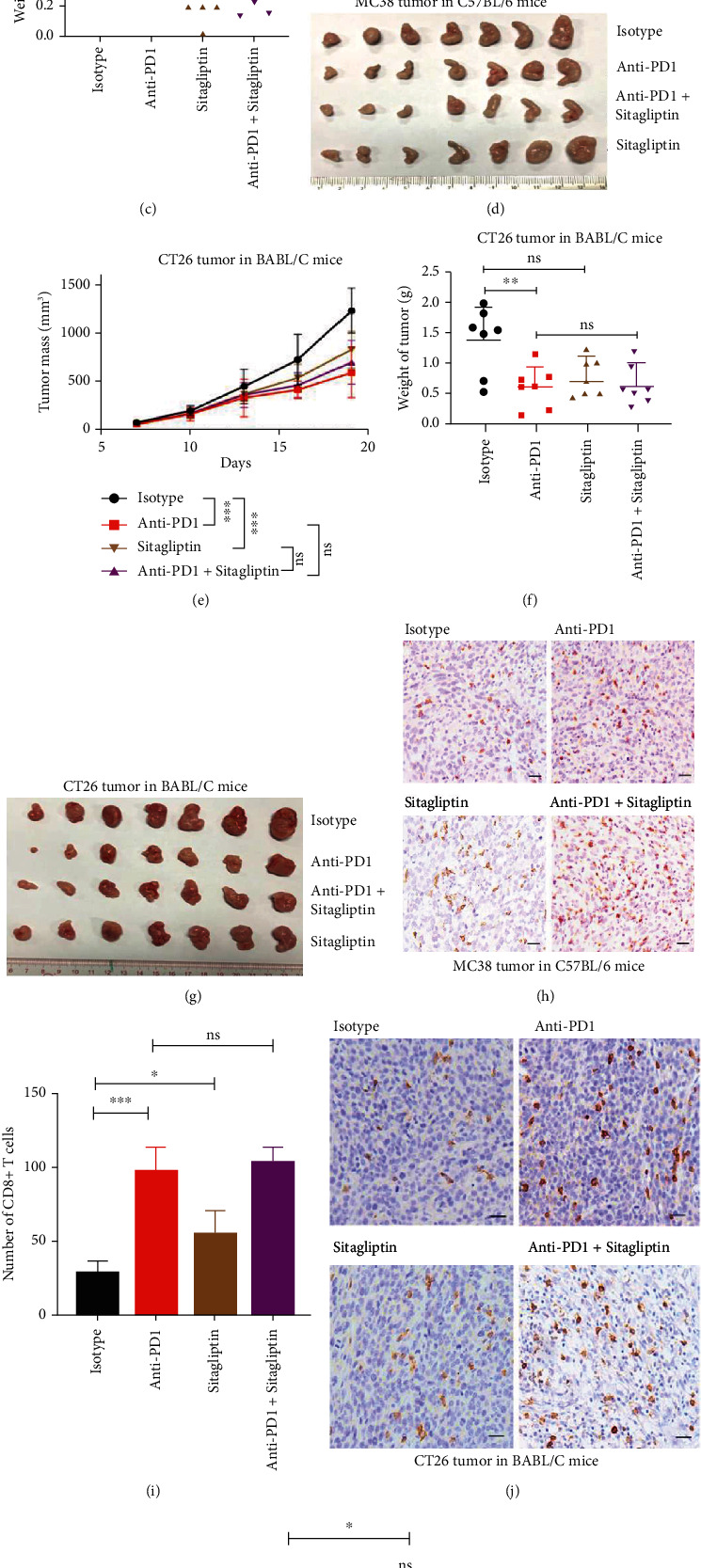
Sitagliptin shows different roles in different tumor models. (a) Schematic overview of experimental design combined for (b)–(k). (b–d) Tumor growth (b) and weight (c) from MC38 tumor-bearing C57BL/6 mice were shown (*n* = 7). (e–g) Tumor growth (e) and weight (g) from CT26 tumor-bearing on BABL/C mice were shown (*n* = 7). (h and i) Sections from MC38 subcutaneous tumors were stained with anti-CD8 antibody by immunohistochemistry (h), and CD8^+^ T cells infiltration were analyzed in (i). (j and k) Sections from CT26 subcutaneous tumors were stained with anti-CD8 antibody by immunohistochemistry (j), and CD8^+^ T cells infiltration were analyzed in (k). Scale bars: 100 *μ*m. Data were analyzed by two-way ANOVA (b and e) and *t*-test (c, f, i, and k). ns: no significance; ^∗^*p* < 0.05, ^∗∗^*p* < 0.01, and ^∗∗∗^*p* < 0.001. Error bars denote for the s.e.m.

Sitagliptin is a dipeptidyl peptidase-4 inhibitors (DPP4-i), which reduces the blood glucose level by increasing the endogenous levels of bioactive incretins, leading to insulin secretion enhancement in a glucose-dependent way [24]. In experiments with a syngeneic mouse colon cancer model (Figure 2(a)), sitagliptin monotherapy and anti-PD1 monotherapy significantly inhibited the growth of subcutaneously grafted MC38 cells in WT C57BL/6 mice, respectively (Figures 2(b)–2(d)). Furthermore, the combination of sitagliptin and anti-PD1 showed a more significant effect on tumor growth inhibition (Figures 2(b)–2(d)). However, although sitagliptin monotherapy and anti-PD1 monotherapy significantly inhibited the growth of subcutaneously grafted CT26 cells in WT Balb/c mice, respectively (Figures 2(e)–2(g)), the combination of sitagliptin and anti-PD1 did not show enhanced effect in suppressing tumor growth (Figures 2(e)–2(g)). Immunohistological examination of the MC38 and CT26 tumors showed that sitagliptin and anti-PD1 significantly increased the number of tumoral infiltrated CD8^+^ T cells, respectively (Figures 2(h)–2(k)), and the combination of sitagliptin and anti-PD1 induced more tumoral infiltrated CD8^+^ T cells in MC38 tumors (Figures 2(h) and 2(i)), but not in CT26 tumors (Figures 2(j) and 2(k)).

In experiments with a syngeneic mouse melanoma cancer model (Figure S2A), sitagliptin alone and anti-PD1 alone had no effect on the tumor growth of subcutaneously grafted B16 cells in WT C57BL/6 mice (Figure S2B-D). The combination of sitagliptin and anti-PD1 had no effect on tumor growth inhibition neither (Figure S2B–D). Immunohistological examination of the B16 tumors showed that sitagliptin had no effect on the number of tumoral infiltrated CD8^+^ T cells (Figure S2E-H).

### 2.3. Metformin Inhibits Tumor Growth and Has No Effect on Tumor Immune Responses to Anti-PD1

**Figure 3:**
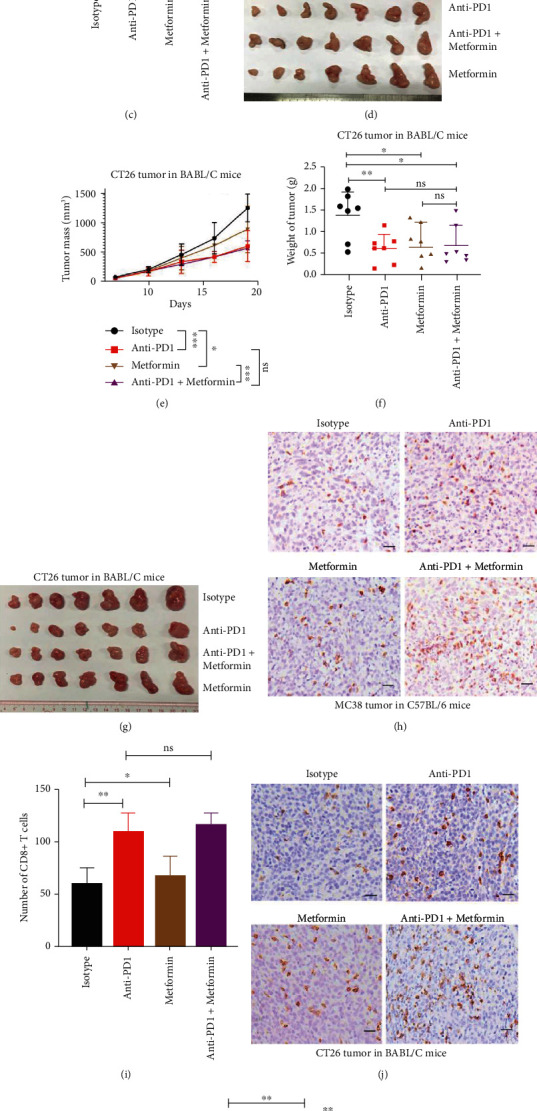
Metformin inhibits colon tumor growth and has no effect on tumor immune responses to anti-PD1. (a) Schematic overview of experimental design combined for (b)–(k). (b–d) Tumor growth (b) and weight (c) from MC38 tumor-bearing C57BL/6 mice were shown (*n* = 7). (e–g) Tumor growth (e) and weight (g) from CT26 tumor-bearing on BABL/C mice were shown (*n* = 7). (h and i) Sections from MC38 subcutaneous tumors were stained with anti-CD8 antibody by immunohistochemistry (h), and CD8^+^ T cells infiltration were analyzed in (i). (j and k) sections from CT26 subcutaneous tumors were stained with anti-CD8 antibody by immunohistochemistry (j), and CD8^+^ T cells infiltration were analyzed in (k). Scale bars: 100 *μ*m. Data were analyzed by two-way ANOVA (b and e) and *t*-test (c, f, i, and k). ns: no significance; ^∗^*p* < 0.05, ^∗∗^*p* < 0.01, and ^∗∗∗^*p* < 0.001. Error bars denote for the s.e.m.

Metformin belongs to the biguanide class of oral hypoglycemic drugs widely used in the treatment of type 2 diabetes mellitus (T2DM). Metformin increases insulin sensitivity which results in increased glucose uptake and decreased gluconeogenesis, thereby reducing serum glucose levels [25]. In experiments with a syngeneic mouse colon cancer model (Figure 3(a)), metformin monotherapy and anti-PD1 monotherapy significantly inhibited the growth of subcutaneously grafted MC38 cells in WT C57BL/6 mice, respectively (Figures 3(b)–3(d)). However, compared with anti-PD1 treatment alone, the combination treatment of metformin and anti-PD1 did not show an improved therapeutic effect on MC38 tumor inhibition (Figures 3(b)–3(d)). Similar results were observed in experiments using mouse colon cancer CT26 cells. Metformin alone and anti-PD1 alone significantly inhibited the growth of subcutaneously grafted CT26 cells in WT Balb/c mice, respectively (Figures 3(e)–3(g)). Compared with anti-PD1 treatment alone, the combination treatment of metformin and anti-PD1 did not show an improved therapeutic effect on CT26 tumor inhibition (Figures 3(e)–3(g)). Immunohistological examination showed that, compared with control, metformin significantly increased the number of infiltrated CD8^+^ T cells in MC38 tumors and CT26 tumors (Figures 3(h)–3(k)). However, compared with anti-PD1 treatment, the combination treatment of metformin and anti-PD1 did not show increased tumoral infiltrated CD8^+^ T cells in MC38 tumors and CT26 tumors (Figures 3(h)–3(k)).

In experiments with a syngeneic mouse melanoma cancer model (Figure S3A), compared with no treatment control, metformin or anti-PD1 alone had no effect on the B16 tumor growth, respectively (Figure S3B-D), and the combination of metformin and anti-PD1 significantly inhibited B16 tumor growth (Figure S3B–D). However, compared with anti-PD1 treatment alone, the combination treatment of metformin and anti-PD1 did not show significant therapeutic effect on B16 tumor inhibition (Figure S3E–G). Immunohistological examination of the B16 tumors showed that metformin significantly increased the number of tumoral infiltrated CD8^+^ T cells in tumors (Figure S3E-F).

### 2.4. Glimepiride Weakens Colon Tumor Immune Responses to Anti-PD1 and Enhances Melanoma Tumor Immune Responses to Anti-PD1

**Figure 4:**
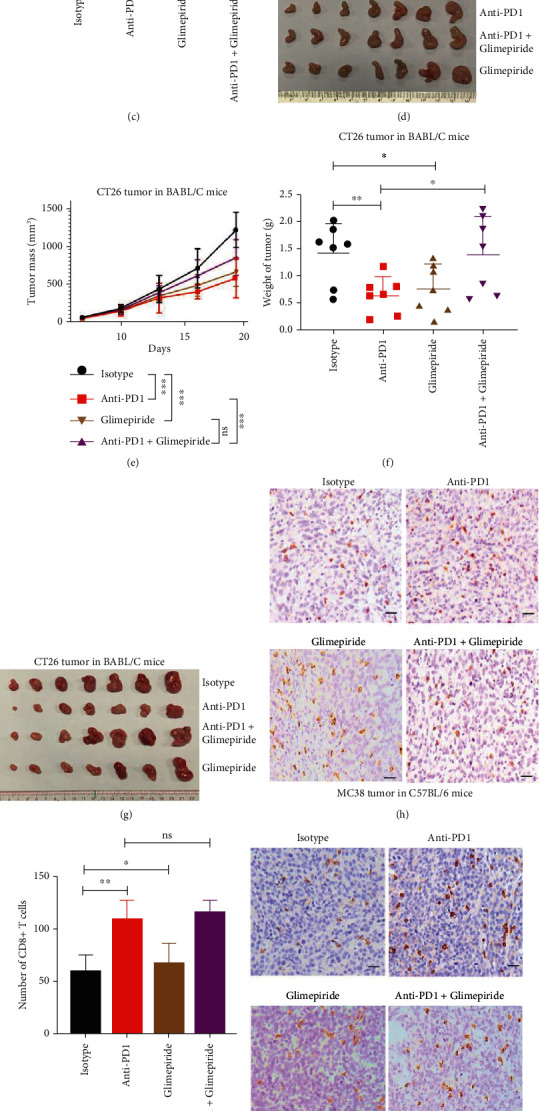
Glimepiride weakens colon tumor immune responses to anti-PD1. (a) Schematic overview of experimental design combined for (b)–(k). (b–d) Tumor growth (b) and weight (c) from MC38 tumor-bearing C57BL/6 mice were shown (*n* = 7). (e–g) Tumor growth (e) and weight (g) from CT26 tumor-bearing on BABL/C mice were shown (*n* = 7). (h and i) Sections from MC38 subcutaneous tumors were stained with anti-CD8 antibody by immunohistochemistry (h), and CD8^+^ T cells infiltration were analyzed in (i). (j and k) Sections from CT26 subcutaneous tumors were stained with anti-CD8 antibody by immunohistochemistry (j), and CD8^+^ T cells infiltration were analyzed in (k). Scale bars: 100 *μ*m. Data were analyzed by two-way ANOVA (b and e) and *t*-test (c, f, i, and k). ns: no significance; ^∗^*p* < 0.05, ^∗∗^*p* < 0.01, and ^∗∗∗^*p* < 0.001. Error bars denote for the s.e.m.

Glimepiride is a sulfonylurea drug that increases the release of insulin by binding to a subunit of potassium ATP-dependent channel known as the sulfonylurea receptor of pancreatic beta cells [26, 27]. In experiments with a syngeneic mouse colon cancer model (Figure 4(a)), glimepiride monotherapy and anti-PD1 monotherapy significantly inhibited the growth of subcutaneously grafted MC38 cells in WT C57BL/6 mice, respectively (Figures 4(b)–4(d)). However, compared with anti-PD1 treatment alone, the combination treatment of glimepiride and anti-PD1 did not show an improved therapeutic effect on MC38 tumor inhibition (Figures 4(b)–4(d)). Glimepiride monotherapy and anti-PD1 monotherapy significantly inhibited the growth of subcutaneously grafted CT26 cells in WT Balb/c mice, respectively (Figures 4(e)–4(g)). However, compared with anti-PD1 treatment alone, the combination treatment of glimepiride and anti-PD1 showed a weakened therapeutic effect on CT26 tumor inhibition (Figures 4(e)–4(g)). Immunohistological examination of the MC38 and CT26 tumors showed that glimepiride alone and anti-PD1 alone significantly increased the number of infiltrated CD8^+^ T cells in tumors, respectively (Figures 4(h)–4(k)). However, compared with anti-PD1 treatment alone, the combination treatment of glimepiride and anti-PD1 did not show increased infiltrated CD8^+^ T cells in MC38 tumors and CT26 tumors (Figures 4(h)–4(k)).

In experiments with a syngeneic mouse melanoma cancer model (Figure S4A), compared with that glimepiride or anti-PD1 alone had no effect on the B16 tumor growth, respectively (Figure S4B-D), the combination of glimepiride and anti-PD1 significantly inhibited B16 tumor growth (Figure S4B–D). Immunohistological examination of the B16 tumors showed that glimepiride significantly increased the number of infiltrated CD8^+^ T cells in tumors (Figure S4E-H).

### 2.5. Pioglitazone Inhibits Tumor Growth and Weakens Tumor Immune Responses to Anti-PD1

**Figure 5:**
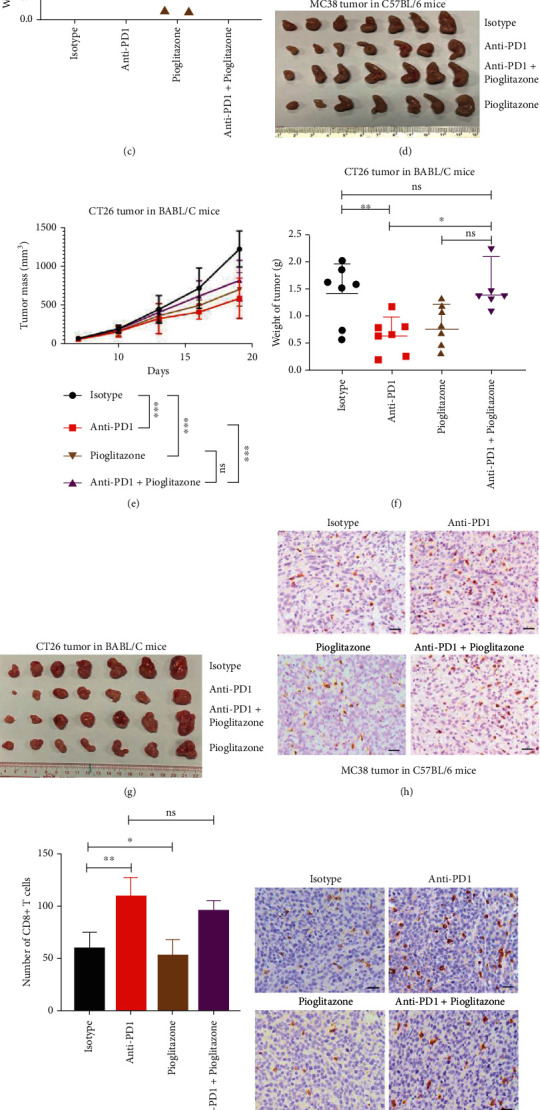
Pioglitazone inhibits colon tumor growth and weakens tumor immune responses to anti-PD1. (a) Schematic overview of experimental design combined for (b)–(k). (b–d) Tumor growth (b) and weight (c) from MC38 tumor-bearing C57BL/6 mice were shown (*n* = 7). (e–g) Tumor growth (e) and weight (g) from CT26 tumor-bearing on BABL/C mice were shown (*n* = 7). (h and i) Sections from MC38 subcutaneous tumors were stained with anti-CD8 antibody by immunohistochemistry (h), and CD8^+^ T cells infiltration were analyzed in (i). (j and k) Sections from CT26 subcutaneous tumors were stained with anti-CD8 antibody by immunohistochemistry (j), and CD8^+^ T cells infiltration were analyzed in (k). Scale bars: 100 *μ*m. Data were analyzed by two-way ANOVA (b and e) and *t*-test (c, f, i, and k). ns: no significance; ^∗^*p* < 0.05, ^∗∗^*p* < 0.01, and ^∗∗∗^*p* < 0.001. Error bars denote for the s.e.m.

Pioglitazone which belongs to thiazolidinedione (TZDs) is a peroxisome proliferator-activated receptor (PPAR)-*γ* agonist that reduces insulin resistance by stimulating lipogenesis, suppressing lipolysis in the adipose tissue and decreasing hepatic triglycerides, visceral fat mass, and activity, thus promoting peripheral insulin sensitivity [28]. In experiments with a syngeneic mouse colon cancer model (Figure 5(a)), anti-PD1, but not pioglitazone, significantly inhibited the growth of subcutaneously grafted MC38 cells in WT C57BL/6 mice (Figures 5(b)–5(d)). However, compared with anti-PD1 treatment alone, the combination treatment of pioglitazone and anti-PD1 showed a weakened therapeutic effect on MC38 tumor inhibition (Figures 5(b)–5(d)). Pioglitazone alone and anti-PD1 alone significantly inhibited the growth of subcutaneously grafted CT26 cells in WT Balb/c mice, respectively (Figures 5(e)–5(g)). However, compared with anti-PD1 treatment alone, the combination treatment of pioglitazone and anti-PD1 show a weakened therapeutic effect on CT26 tumor inhibition (Figures 5(e)–5(g)). Immunohistological examination of the MC38 and CT26 tumors showed that anti-PD1 significantly increased the number of infiltrated CD8^+^ T cells in tumors, while pioglitazone decreased the number of infiltrated CD8^+^ T cells in tumors (Figures 5(h)–5(k)). Furthermore, compared with anti-PD1 treatment, the combination treatment of pioglitazone and anti-PD1 decreased infiltrated CD8^+^ T cells in CT26 tumors, significantly (Figures 5(h)–5(k)).

In experiments with a syngeneic mouse melanoma cancer model (Figure S5A), compared with that anti-PD1 alone had no effect on the B16 tumor growth, pioglitazone alone significantly inhibited B16 tumor growth (Figure S5B-D). However, the combination of pioglitazone and anti-PD1 had no effect on the B16 tumor growth (Figure S5B–D). Immunohistological examination of the B16 tumors showed that pioglitazone and anti-PD1 had no effect on the number of infiltrated CD8^+^ T cells in B16 tumors (Figure S5E-H).

### 2.6. Insulin Promotes Tumor Growth and Weakens Tumor Immune Responses to Anti-PD1

**Figure 6:**
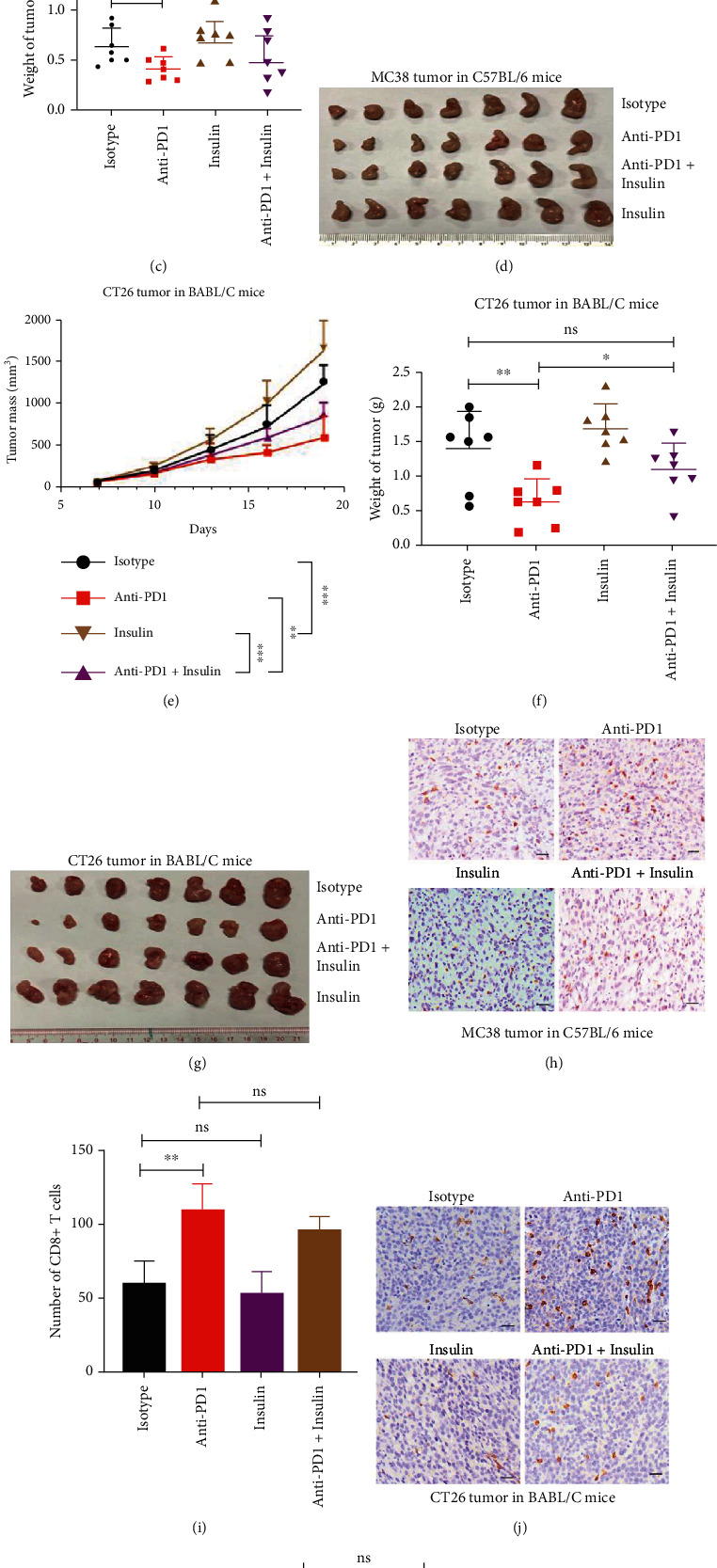
Insulin promotes colon tumor growth and weakens tumor immune responses to anti-PD1. (a) Schematic overview of experimental design combined for (b)–(k). (b–d) Tumor growth (b) and weight (c) from MC38 tumor-bearing C57BL/6 mice were shown (*n* = 7). (e–g) Tumor growth (e) and weight (g) from CT26 tumor-bearing on BABL/C mice were shown (*n* = 7). (h and i) Sections from MC38 subcutaneous tumors were stained with anti-CD8 antibody by immunohistochemistry (h) and CD8^+^ T cells infiltration were analyzed in (i). (j and k) Sections from CT26 subcutaneous tumors were stained with anti-CD8 antibody by immunohistochemistry (j), and CD8^+^ T cells infiltration were analyzed in (k). Scale bars: 100 *μ*m. Data were analyzed by two-way ANOVA (b and e) and *t*-test (c, f, i, and k). ns: no significance; ^∗^*p* < 0.05, ^∗∗^*p* < 0.01, and ^∗∗∗^*p* < 0.001. Error bars denote for the s.e.m.

Insulin is produced by pancreatic *β* cells that is essential especially for the metabolism of carbohydrates and the regulation of glucose levels in the blood and that when insufficiently produced results in diabetes mellitus. Insulin injection is usually used when oral hypoglycemic drugs are not ideal for blood glucose control [29]. In experiments with a syngeneic mouse colon cancer model (Figure 6(a)), anti-PD1 significantly inhibited the growth of subcutaneously grafted MC38 cells in WT C57BL/6 mice, while insulin promoted MC38 tumor growth (Figures 6(b)–6(d)). Compared with anti-PD1 treatment alone, the combination treatment of insulin and anti-PD1 showed a weakened therapeutic effect on MC38 tumor inhibition (Figures 6(b)–6(d)). Similar results were observed in experiments using mouse colon cancer CT26 cells. Anti-PD1 significantly inhibited CT26 tumor growth, while insulin promoted CT26 tumor growth (Figures 6(e)–6(g)). Compared with anti-PD1 treatment alone, the combination treatment of insulin and anti-PD1 showed a weakened therapeutic effect on CT26 tumor inhibition (Figures 6(e)–6(g)). Immunohistological examination of the MC38 and CT26 tumors showed that anti-PD1 significantly increased the number of infiltrated CD8^+^ T cells, while insulin significantly decreased the number of tumoral infiltrated CD8^+^ T cells (Figures 6(h)–6(k)). Compared with anti-PD1 alone, the combination of insulin and anti-PD1 decreased the number of infiltrated CD8^+^ T cells in CT26 tumors (Figures 6(h) and 6(i)).

In experiments with a syngeneic mouse melanoma cancer model (Figure S6A), insulin or anti-PD1 alone had no effect on the tumor growth (Figure S6B-D). The combination of insulin and anti-PD1 had no effect on tumor growth inhibition either (Figure S6B–D). Immunohistological examination of the B16 tumors showed that anti-PD1 had no effect on the number of tumoral infiltrated CD8^+^ T cells, while insulin decreased the number of tumoral infiltrated CD8^+^ T cells (Figure S6E-H).

### 2.7. Comparison of Six Antidiabetic Drugs

For each tumor cell line, all of the in vivo experiments share the same isotype control and anti-PD1 controls, and all of the tumor tissues were collected at the same time point. We put all the data of each tumor cell line in one table and in one figure to compare the effect of the six antidiabetic drugs on tumor inhibition (Table S1). Acarbose combined with anti-PD1 has the best effect in the treatment of tumors (Figure S7-S9). We analyzed the correlation between the key genes of diabetes and the survival rate of colorectal cancer using TCGA database and found that there was no significant correlation between the expression of IGF1R/IGF2R and the survival rate of colorectal cancer. The expression of PPARG was negatively correlated with the survival rate of colorectal cancer. But the expression of IGF1R, IGF2R, and PPARG was negatively correlated with the number of infiltrated CD8+ T cells in colorectal cancer (Figure S10); the related antidiabetic drugs may produce negative effect on anti-PD1 tumor inhibition.

We further performed the in vitro experiments to detect the inhibitory effect of each antidiabetic drugs on CT26 cell proliferation. The results showed that acarbose, sitagliptin, and metformin had no effect on the proliferation of tumor cells; pioglitazone significantly inhibited CT26 cell proliferation; glimepiride and insulin significantly promoted CT26 cell proliferation (Figure S11).

We also monitored the blood glucose of mice treated with acarbose and insulin and find that the effect of acarbose and insulin on anti-PD1 tumor inhibition was not related to blood glucose (Figure S12). In addition, we collected the data about the mice weight in the day of MC38 tumor harvested; the mice weight of insulin, anti-PD1 + insulin, anti-PD1 + glimepiride group significantly increased, while the other groups showed no significant difference compared to the isotype (Figure S13).

## 3. Discussion

Immunotherapy has become a powerful clinical strategy for the treatment of cancer. Considering the large number of cancer patients codiagnosed with diabetes, it is important to understand whether the antidiabetic drugs have any effect on tumor immunotherapy. It has at least two advantages: (1) it can help diabetic patients choose right antidiabetic drugs when receiving tumor immunotherapy. (2) It may provide new therapeutic strategies based on the combination of antidiabetic drugs and tumor immunotherapy for cancer patients with or without diabetic. We studied the effects of 6 common antidiabetic drugs on tumor inhibition of anti-PD1 immune checkpoint inhibitor, including acarbose, sitagliptin, metformin, glimepiride, pioglitazone, and insulin. We found that different antidiabetic drugs have different effects in different tumor, which may improve, reduce, or have a neutral effect on the tumor inhibition of anti-PD1. Specifically, acarbose and sitagliptin synergize with anti-PD1 in colon and melanoma tumor treatment. Metformin has no effect on tumor inhibition of anti-PD1. Glimepiride, pioglitazone, and insulin weaken the effect of anti-PD1 in tumor treatment.

Acarbose has been widely used clinically to prevent postprandial hyperglycemia. Retrospective cohort study and meta-analysis report that acarbose reduces the risk of colorectal cancer [30] and lung cancer [31]. Compared with other antidiabetic drugs, patients with acarbose have the lowest risk of aggressive papillary thyroid tumor growth [32]. In animal models, acarbose improves lifespan and reduces tumors in APC knockout and Apc^+/Min^ mouse models [33, 34]. Combining acarbose with anti-PD1 or rapamycin significantly reduces lung metastases of kidney cancer [35]. In this study, we demonstrated that acarbose treatment alone significantly inhibited the growth of colorectal tumor and melanoma. Furthermore, acarbose increased the number of infiltrated CD8^+^ T cells in tumors and improved the therapeutic effect of anti-PD1 on colorectal tumors and melanoma, significantly. These results show that acarbose has excellent antitumor growth and antitumor immune escape effects. We strongly suggest acarbose as treatment of choice to control hyperglycemia in patients codiagnosed with diabetes and cancers when receiving anti-PD1 treatment. Even more, the combination of acarbose and anti-PD1 can further be used as a new strategy for the treatment of various tumors, regardless of whether the patient is diabetes. Further clinical trials should be carried out accordingly.

Sitagliptin is the first DPP4 inhibitor approved for clinical use in 2006 and as the two- or three-line treatment of patients with T2DM [36]. Retrospective cohort studies show that sitagliptin treatment reduces breast cancer risk in female patients with T2DM [37] and improves overall survival of diabetic patients with colorectal or lung cancer surgery [38]. In vitro and in vivo studies show that sitagliptin decreases CRC cell and ovarian cancer cells motility [39, 40], inhibits gastric cancer cells proliferation [41], and prevents colon cancer in rats and mice [42, 43]. Consistent with our results that sitagliptin increased the number of tumoral infiltrated CD8+ T cells and improved the therapeutic effect of anti-PD1 on colorectal tumors and melanoma in this study, recent study shows that sitagliptin upregulates CXCL10 to increase CD8^+^ T lymphocyte infiltration and acts synergistically with anti-PD1 treatment for HCC and melanoma in mouse model [44, 45]. The retrospective cohort study shows that tissues from HCC patients with diabetes undergoing sitagliptin treatment had a higher level of CD8^+^ T lymphocyte infiltration than tissues from HCC patients with diabetes without sitagliptin treatment, which further supported that sitagliptin could elevate the efficacy of anti-PD1 antibody therapy [45]. In addition, sitagliptin can also increase the recruitment of eosinophils into mouse models of hepatocellular carcinoma (HCC) and breast cancer to control tumor growth [46]. Although it is reported that sitagliptin is significantly associated with a higher risk of pancreatic cancer [47], sitagliptin is still recommended as an antidiabetic drug in other cancer patients with diabetes, and it can further be used in clinical trials to verify its role in combination with anti-PD1 in various tumor treatments.

Metformin is a safe, widely used, first-line oral medication to treat T2DM. Previous large case-control studies revealed that metformin treatment reduced the incidence of various cancer types [13, 48, 49]. Metformin can inhibit tumor in many ways, including regulating the PI3K (phosphatidylinositol 3-kinase), mammalian target of rapamycin (mTOR), AMP-activated protein kinase, and MAPK (mitogen-activated protein kinase) signaling pathways [50, 51]. Recent study reports that metformin increases cytotoxic T lymphocyte (CTL) activity against cancer cells, and the combination of metformin and CTLA4 blockade increases the efficacy of immunotherapy [52]. Our study showed that metformin monotherapy and anti-PD1 monotherapy significantly inhibits colon tumor growth and CD8^+^ T cells tumoral infiltration, respectively. However, metformin and anti-PD1 had no synergistic effect on antitumor. We suggest that metformin can be used as an antidiabetic drug in cancer patients with diabetes under anti-PD1 treatment, because metformin does not inhibit the antitumor effect of anti-PD1.

Glimepiride, classified as a third-generation sulfonylurea, is an oral antidiabetic drug for the treatment of T2DM [53]. The effect of glimepiride on tumors is controversial. A case-control study showed that glimepiride did not increase overall cancer risk [54], whereas some studies reported that glimepiride increased the risk for colorectal cancer (CRC) [55] and hepatocellular carcinoma (HCC) in type 2 diabetic patients [56]. It has also been reported that glimepiride can inhibit MCF-7 breast cancer cell proliferation [57]. In a glioblastoma multiforme (GBM) orthotopic xenograft mouse model, combining radiotherapy and glimepiride significantly reduced GBM growth and improved survival [58]. Our study shows that glimepiride monotherapy induces increased tumoral CD8^+^ T cells infiltration and inhibits colon tumor growth. However, glimepiride decreases the therapeutic effect of anti-PD1 on CT26 tumor growth. Contrary to colon cancer, glimepiride or anti-PD1 monotherapy has no effect on melanoma growth, but the combination of glimepiride and anti-PD1 significantly inhibits melanoma growth. These results indicate that the effect of glimepiride on different tumors is different. Therefore, we suggest that glimepiride can be used as antidiabetic drug in melanoma but not colon cancer patients with diabetes under anti-PD1 treatment.

Pioglitazone, as the only insulin sensitizer available, is broadly used for glycemic control in patients with T2DM. Some retrospective analyses reported that pioglitazone is associated with increased risk of bladder cancer [59–61], which is also suggested in a rat bladder cancer model [62]. Pioglitazone is also likely to be a causal risk factor for prostate cancer and pancreatic cancer [63]. Another cohort study showed that pioglitazone is associated with increased incidence of melanoma and non-Hodgkin lymphoma and decreased risk of renal cancer [64]. Our study shows that pioglitazone monotherapy inhibits colon tumor and melanoma growth. However, glimepiride decreases the number of tumoral infiltrated CD8^+^ T cells and weakens the therapeutic effect of anti-PD1 on colon tumor growth inhibition. The combination of glimepiride and anti-PD1 also failed to inhibit melanoma growth. Therefore, we do not suggest glimepiride as antidiabetic drug in colon cancer and melanoma patients with diabetes under anti-PD1 treatment.

Insulin is always used when hyperglycemia cannot be adequately controlled by oral antidiabetic drugs, and approximately half of T2DM patients receive insulin therapy for ideal glucose control [29]. Insulin is a major regulator of cell metabolism and also a growth factor. The role of insulin and its receptor in tumorigenesis is supported by clinical evidences and laboratory models [65]. Cohort studies and meta-analysis showed that insulin exposure is associated with an increased risk of cancer in the pancreas, liver, kidney, stomach, respiratory system, breast, and colon [66–70]. Our study shows that insulin promotes colon tumor growth, decreases the number of tumoral infiltrated CD8^+^ T cells, and weakens the therapeutic effect of anti-PD1 on colon tumor growth inhibition. More clinical evidence is needed to help form an expert consensus on whether insulin should be used cautiously in tumor patients receiving anti-PD1 antibody therapy.

In conclusion, we suggest that in the treatment of colon cancer with anti-PD1, diabetic patients should take acarbose and sitagliptin to control blood glucose, which can improve the inhibitory effect of anti-PD1 colon tumor. Glimepiride, pioglitazone, and insulin are not recommended because they may reduce the colon tumor inhibitory effect of anti-PD1. Metformin has no effect on colon tumor inhibition of anti-PD1 and can be taken. In the treatment of melanoma with anti-PD1, diabetic patients should take acarbose, metformin, and glimepiride to control blood glucose, which can improve the inhibitory effect of anti-PD1 on melanoma. Sitagliptin, pioglitazone, and insulin have no effect on the melanoma tumor inhibition of anti-PD1. Clinical trials are needed for further validation. More research groups are encouraged to study other antidiabetic drugs and various tumor types. We believe that many cancer patients will benefit from these studies.

## 4. Materials and Methods

### 4.1. Cell Lines

Murine colon MC38 cells and CT26 cells are gift from Professor Wei Yang of the Southern Medical University, and murine melanoma B16F10 cells were originally obtained from the American Type Culture Collection (ATCC). All of them were cultured in DMEM (Gibco) supplemented with 10% fetal bovine serum (FBS). And incubated at 37°C under a humidified atmosphere with 5% CO2. All cell lines were routinely tested for mycoplasma, the results of which were negative.

### 4.2. Animal Study

All experiments related to animal studies were endorsed by the Animal Care and Use Committee of the Southern Medical University, and all procedures followed the NIH guidelines for animal handling.

For tumorigenesis assays, 0.5 − 1 × 10^6^ cells (MC38 and CT26) or 0.1 − 0.5 × 10^6^ cells (B16F10) per mouse were subcutaneously injected into the right dorsal flanks of female C57BL/6 J or Balb/c mice (5–6 weeks of age, 18–20 g) which were obtained from the Guangdong Medical Laboratory Animal Center, China. The mice were sacrificed when tumor approached nearly 1500 mm3 or obvious ulceration happened at approximately 7 to 8 weeks. The tumors were excised and placed in 10% neutral buffered formalin for 24 h. The tumor size was measured using a slide caliper, and the tumor volume was determined by the following formula: 0.5 × *A* × *B*2, where *A* represents the diameter of the base of the tumor and *B* represents the corresponding perpendicular value.

For drug treatment, on days 6-7, when there were 1–10 mm^2^ palpable tumors, mice were started on either 0.25 mg anti-PD1 or hamster IgG isotype control (BioXCell), injected every 2 days; total dose is 1 mg per mouse intraperitoneally, and the antidiabetic drug metformin (200 mg/kg, TargetMol), pioglitazone (20 mg/kg, TargetMol), sitagliptin (20 mg/kg, TargetMol) or PBS, and acarbose (500 mg/kg, TargetMol) took intragastrical administration every day; glimepiride (5 mg/kg, TargetMol) and insulin (0.035 mg per mouse, TargetMol)) or PBS took intraperitoneal injection every 2 days. This drug regimen was based on a review of the literature and the book of *Experimental Methodology of Pharmacology* edited by Xu Shu Yun.

### 4.3. Immunohistochemistry

The tumor tissues were cut into 4-*μ*m-thick sections, baked at 65°C for 1 h, and dewaxed by dimethylbenzene and alcohol. The sections were deparaffinized with xylene and treated with 3% hydrogen peroxide to attenuate endogenous peroxidase activity. Next, the sections were submerged in citrate buffer for antigen retrieval and incubated with 10% bovine serum albumin (BSA) to block nonspecific binding. Primary antibodies against CD8 (1 : 1000, ab209775, ABCAM) were used according to the manufacturer's instructions. The sections were incubated with DAB and hematoxylin. Finally, the sections were taken photo and count the positive cell number.

### 4.4. Statistical Analyses

Statistical parameters are all shown in figure legends. Statistical analysis was performed using nonparametric two-tailed *t*-test or two-way ANOVA in by SPSS 25.0 for Mac. Unless specially described, error bars stand for standard error of the mean. ns means no significance; ^∗^*p* < 0.05; ^∗∗^*p* < 0.01; and ^∗∗∗^*p* < 0.001.

## Data Availability

All data are available in the main text or the supplementary materials.

## Additional Points

*One Sentence Summary.* Acarbose significantly inhibited tumor growth and further enhanced the therapeutic effect of anti-PD1.

## Supplementary Materials

Supplementary Materials Fig. S1: acarbose inhibits melanoma tumor growth and enhances tumor immune responses to anti-PD1. Figure S2: sitagliptin has no effects on melanoma tumor growth and tumor immune responses to anti-PD1. Figure S3: metformin has no effects on melanoma tumor growth and tumor immune responses to anti-PD1. Figure S4: glimepiride enhances melanoma tumor immune responses to anti-PD1. Figure S5: pioglitazone inhibits melanoma tumor growth, but anti-PD1 weakens tumor inhibition of pioglitazone. Figure S6: insulin has no effects on melanoma tumor growth and tumor immune responses to anti-PD1. Figure S7: compare the effect of the six antidiabetic drugs on MC38 tumor inhibition. Figure S8: compare the effect of the six antidiabetic drugs on CT26 tumor inhibition. Figure S9: compare the effect of the six antidiabetic drugs on B16F10 tumor inhibition. Figure S10: the expression of IGF1R, IGF2R, and PPARG was negatively correlated with the number of infiltrated CD8+ T cells in colorectal cancer. Figure S11: the inhibitory effect of each antidiabetic drugs on CT26 cell proliferation. Figure S12: the effect of acarbose and insulin on anti-PD1 tumor inhibition was not related to blood glucose. Figure S13: the mice weight of each group in the day of MC38 tumor harvested. Table.S1: the weight of tumor after different Intervention
