# Supplementary material for: The Effects of 6 Common Antidiabetic Drugs on Anti-PD1 Immune Checkpoint Inhibitor in Tumor Treatment
Source: J Immunol Res. 2022 Aug 18;2022:2651790. doi: 10.1155/2022/2651790 (PMC9410852; doi:10.1155/2022/2651790)
Supplement: Supplementary Materials — Fig. S1: acarbose inhibits melanoma tumor growth and enhances tumor immune responses to anti-PD1. Figure S2: sitagliptin has no effects on melanoma tumor growth and tumor immune responses to anti-PD1. Figure S3: metformin has no effects on melanoma tumor growth and tumor immune responses to anti-PD1. Figure S4: glimepiride enhances melanoma tumor immune responses to anti-PD1. Figure S5: pioglitazone inhibits melanoma tumor growth, but anti-PD1 weakens tumor inhibition of pioglitazone. Figure S6: insulin has no effects on melanoma tumor growth and tumor immune responses to anti-PD1. Figure S7: compare the effect of the six antidiabetic drugs on MC38 tumor inhibition. Figure S8: compare the effect of the six antidiabetic drugs on CT26 tumor inhibition. Figure S9: compare the effect of the six antidiabetic drugs on B16F10 tumor inhibition. Figure S10: the expression of IGF1R, IGF2R, and PPARG was negatively correlated with the number of infiltrated CD8+ T cells in colorectal cancer. Figure S11: the inhibitory effect of each antidiabetic drugs on CT26 cell proliferation. Figure S12: the effect of acarbose and insulin on anti-PD1 tumor inhibition was not related to blood glucose. Figure S13: the mice weight of each group in the day of MC38 tumor harvested. Table.S1: the weight of tumor after different Intervention [file 2651790.f1.zip › Figure S7.pdf]

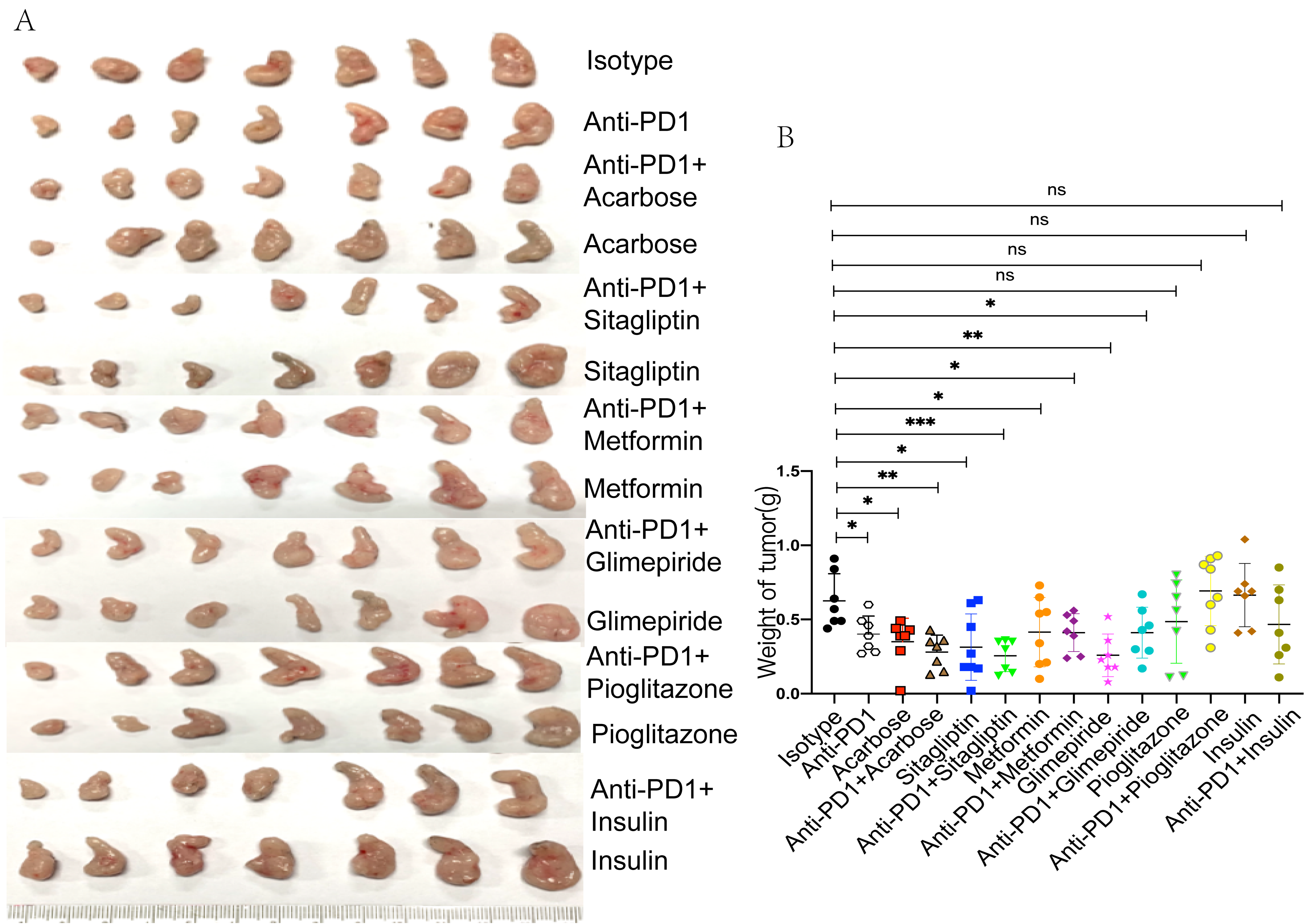

A
Isotype
Anti-PD1
Anti-PD1+ Acarbose
Acarbose
Anti-PD1+ Sitagliptin
Sitagliptin
Anti-PD1+ Metformin
Metformin
Anti-PD1+ Glimepiride
Glimepiride
Anti-PD1+ Pioglitazone
Pioglitazone
Anti-PD1+ Insulin
Insulin
B
ns
ns
ns
ns
*
**
*
*
***
*
**
*
*
Weight of tumor(g)
1.5
1.0
0.5
0.0
Isotype
Anti-PD1
Acarbose
Anti-PD1+Acarbose
Sitagliptin
Anti-PD1+Sitagliptin
Metformin
Anti-PD1+Metformin
Glimepiride
Anti-PD1+Glimepiride
Pioglitazone
Anti-PD1+Pioglitazone
Insulin
Anti-PD1+Insulin
