# Supplementary material for: The Effects of 6 Common Antidiabetic Drugs on Anti-PD1 Immune Checkpoint Inhibitor in Tumor Treatment
Source: J Immunol Res. 2022 Aug 18;2022:2651790. doi: 10.1155/2022/2651790 (PMC9410852; doi:10.1155/2022/2651790)
Supplement: Supplementary Materials — Fig. S1: acarbose inhibits melanoma tumor growth and enhances tumor immune responses to anti-PD1. Figure S2: sitagliptin has no effects on melanoma tumor growth and tumor immune responses to anti-PD1. Figure S3: metformin has no effects on melanoma tumor growth and tumor immune responses to anti-PD1. Figure S4: glimepiride enhances melanoma tumor immune responses to anti-PD1. Figure S5: pioglitazone inhibits melanoma tumor growth, but anti-PD1 weakens tumor inhibition of pioglitazone. Figure S6: insulin has no effects on melanoma tumor growth and tumor immune responses to anti-PD1. Figure S7: compare the effect of the six antidiabetic drugs on MC38 tumor inhibition. Figure S8: compare the effect of the six antidiabetic drugs on CT26 tumor inhibition. Figure S9: compare the effect of the six antidiabetic drugs on B16F10 tumor inhibition. Figure S10: the expression of IGF1R, IGF2R, and PPARG was negatively correlated with the number of infiltrated CD8+ T cells in colorectal cancer. Figure S11: the inhibitory effect of each antidiabetic drugs on CT26 cell proliferation. Figure S12: the effect of acarbose and insulin on anti-PD1 tumor inhibition was not related to blood glucose. Figure S13: the mice weight of each group in the day of MC38 tumor harvested. Table.S1: the weight of tumor after different Intervention [file 2651790.f1.zip › Table.S1..pdf]

Table.S1. The weight of tumor after different Intervention

Intervention	Weight of tumor(g)		
Drug	MC38(s.c.)	CT26 (s.c.)	B16F10(s.c)
Isotype(i.p.), (mean+std)	0.63+0.18	1.38+0.55	1.94+0.67
Anti-PD1(i.p.), (mean+std)	0.40+0.12	0.61+0.33	1.64+0.51
Acarbose(i.g.), (mean+std)	0.35+0.16(green ▲)	0.86+0.59(blue ▲)	1.24+0.36(green ▲)
Glimepiride(i.p.), (mean+std)	0.25+0.14(green ▲)	0.73+0.46(green ▲)	1.56+0.51(blue ▲)
Pioglitazone(i.g.), (mean+std)	0.48+0.28(blue ▲)	0.62+0.32(green ▲)	1.04+0.77(green ▲)
Sitagliptin(i.g.), (mean+std)	0.31+0.22(green ▲)	0.77+0.32(blue ▲)	1.46+0.45(blue ▲)
Metformin(i.g.), (mean+std)	0.41+0.23(green ▲)	0.79+0.37(green ▲)	1.57+0.43(blue ▲)
Insulin(i.p.) , (mean+std)	0.66+0.21(blue ▲)	1.68+0.34(blue ▲)	2.08+0.36(blue ▲)
Anti-PD1+ Acarbose,(mean+std)	0.28+0.11(green ●)	0.29+0.14 (green ●)	1.04+0.46(green ●)
Anti-PD1+ Glimepiride,(mean+std)	0.41+0.17(blue ●)	1.36+0.70(green ●)	0.97+0.34(green ●)
Anti-PD1+ Pioglitazone,(mean+std)	0.70+0.25(red ●)	1.04+0.35(green ●)	1.99+0.77(blue ●)
Anti-PD1+ Sitagliptin,(mean+std)	0.25+0.10(green ●)	0.70+0.37(blue ●)	1.38+0.32(blue ●)
Anti-PD1+ Metformin,(mean+std)	0.41+0.12(blue ●)	0.77+0.47(blue ●)	1.26+0.44(blue ●)
Anti-PD1+ Insulin,(mean+std)	0.46+0.26(blue ●)	1.08+0.38(red ●)	1.99+0.77(blue ●)

green ▲: Drugs exert Inhibition effect on tumor;
blue ▲: No significant effect on tumor;
red ▲: Promoting effect on tumor.

green ●: Enhanced effect of Anti-PD1 on tumor;
blue ●: No significant effect of Anti-PD1 on tumor;
red ●: Reverse effect of Anti-PD1 on tumor.
