# Supplementary material for: The Effects of 6 Common Antidiabetic Drugs on Anti-PD1 Immune Checkpoint Inhibitor in Tumor Treatment
Source: J Immunol Res. 2022 Aug 18;2022:2651790. doi: 10.1155/2022/2651790 (PMC9410852; doi:10.1155/2022/2651790)
Supplement: Supplementary Materials — Fig. S1: acarbose inhibits melanoma tumor growth and enhances tumor immune responses to anti-PD1. Figure S2: sitagliptin has no effects on melanoma tumor growth and tumor immune responses to anti-PD1. Figure S3: metformin has no effects on melanoma tumor growth and tumor immune responses to anti-PD1. Figure S4: glimepiride enhances melanoma tumor immune responses to anti-PD1. Figure S5: pioglitazone inhibits melanoma tumor growth, but anti-PD1 weakens tumor inhibition of pioglitazone. Figure S6: insulin has no effects on melanoma tumor growth and tumor immune responses to anti-PD1. Figure S7: compare the effect of the six antidiabetic drugs on MC38 tumor inhibition. Figure S8: compare the effect of the six antidiabetic drugs on CT26 tumor inhibition. Figure S9: compare the effect of the six antidiabetic drugs on B16F10 tumor inhibition. Figure S10: the expression of IGF1R, IGF2R, and PPARG was negatively correlated with the number of infiltrated CD8+ T cells in colorectal cancer. Figure S11: the inhibitory effect of each antidiabetic drugs on CT26 cell proliferation. Figure S12: the effect of acarbose and insulin on anti-PD1 tumor inhibition was not related to blood glucose. Figure S13: the mice weight of each group in the day of MC38 tumor harvested. Table.S1: the weight of tumor after different Intervention [file 2651790.f1.zip › Table.S1.docx]

**Table.S1. The weight of tumor after different Intervention**

**：Enhanced effect of Anti-PD1 on tumor;**

**: No significant effect of Anti-PD1 on tumor;**

**: Reverse effect of Anti-PD1 on tumor.**

| **Intervention** | **Weight of tumor(g)** | | |
| --- | --- | --- | --- |
| **Drug** | **MC38(s.c.)** | **CT26 (s.c.)** | **B16F10(s.c)** |
| **Isotype****(i.p.)****,**  **(mean+std)** | **0.63+0.18** | **1.38+0.55** | **1.94+0.67** |
| **Anti-PD1****(i.p.), (mean+std)** | **0.40+0.12** | **0.61+0.33** | **1.64+0.51** |
| **Acarbose****(i.g.), (mean+std)** | **0.35+0.16( )**  c | **0.86+0.59( )** | **1.24+0.36( )** |
| **Glimepiride(i.p.), (mean+std)** | **0.25+0.14( )** | **0.73+0.46( )** | **1.56+0.51( )** |
| **Pioglitazone****(i.g.), (mean+std)** | **0.48+0.28( )** | **0.62+0.32( )** | **1.04+0.77( )** |
| **Sitagliptin(i.g.), (mean+std)** | **0.31+0.22( )** | **0.77+0.32( )** | **1.46+0.45( )** |
| **Metformin****(i.g.), (mean+std)** | **0.41+0.23( )** | **0.79+0.37( )** | **1.57+0.43( )** |
| **Insulin(i.p.) ,**  **(mean+std)** | **0.66+0.21****( )** | **1.68+0.34( )** | **2.08+0.36( )** |
| **Anti-PD1+**  **Acarbose,(mean+std)** | **0.28+0.11****( )** | **0.29+0.14** **( )** | **1.04+0.46( )** |
| **Anti-PD1+**  **Glimepiride,(mean+std)** | **0.41+0.17( )** | **1.36+0.70( )** | **0.97+0.34( )** |
| **Anti-PD1+**  **Pioglitazone,(mean+std)** | **0.70+0.25( )** | **1.04+0.35( )** | **1.99+0.77( )** |
| **Anti-PD1+**  **Sitagliptin,(mean+std)** | **0.25+0.10( )** | **0.70+0.37( )** | **1.38+0.32( )** |
| **Anti-PD1+**  **Metformin,(mean+std)** | **0.41+0.12( )** | **0.77+0.47( )** | **1.26+0.44( )** |
| **Anti-PD1+**  **Insulin,(mean+std)** | **0.46+0.26( )** | **1.08+0.38( )** | **1.99+0.77( )** |

**：Drugs exert Inhibition effect on tumor;**

**: No significant effect on tumor;**

**: Promoting effect on tumor.**
