# Supplementary figures and images for: The Effects of 6 Common Antidiabetic Drugs on Anti-PD1 Immune Checkpoint Inhibitor in Tumor Treatment
Source: J Immunol Res. 2022 Aug 18;2022:2651790. doi: 10.1155/2022/2651790 (PMC9410852; doi:10.1155/2022/2651790)

A

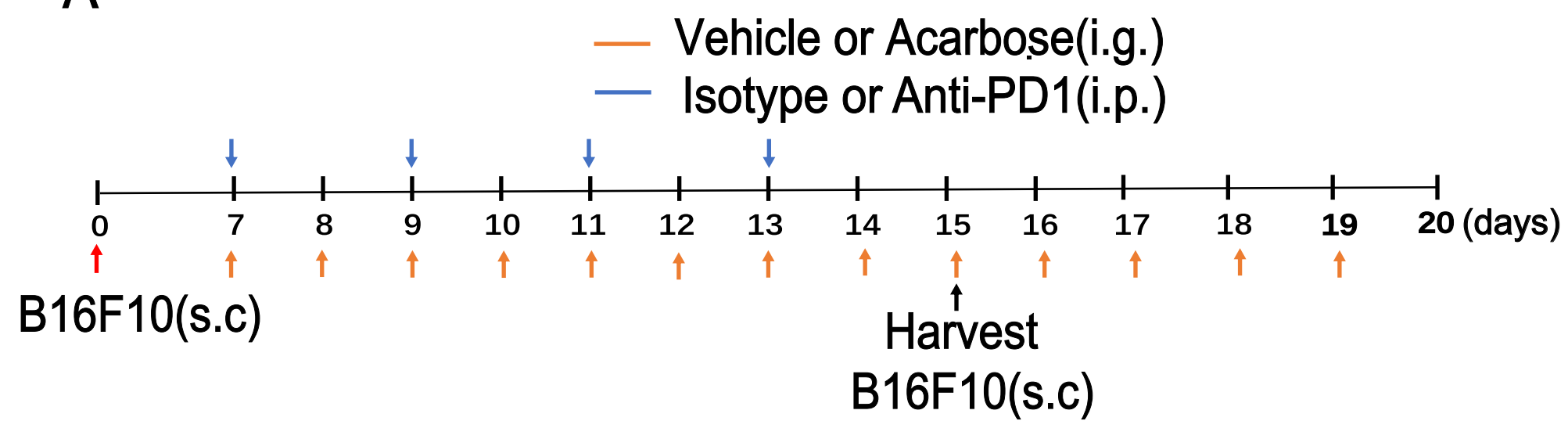

B16F10 tumor in C57BL/6 mice

B

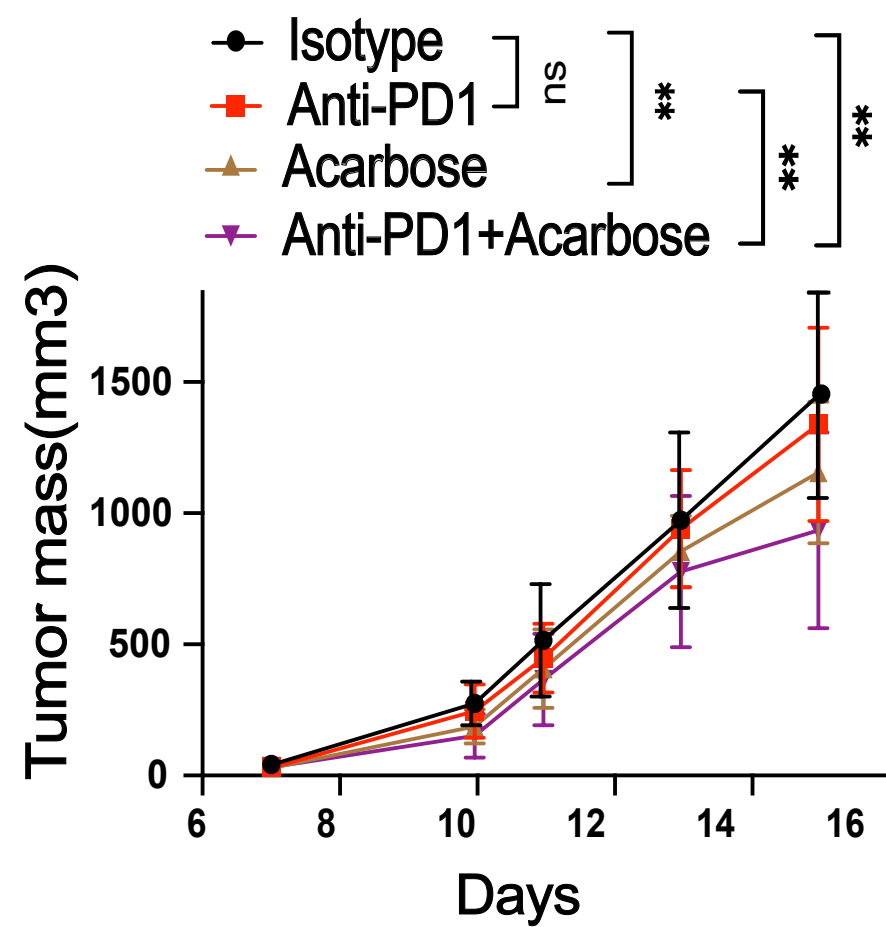

C

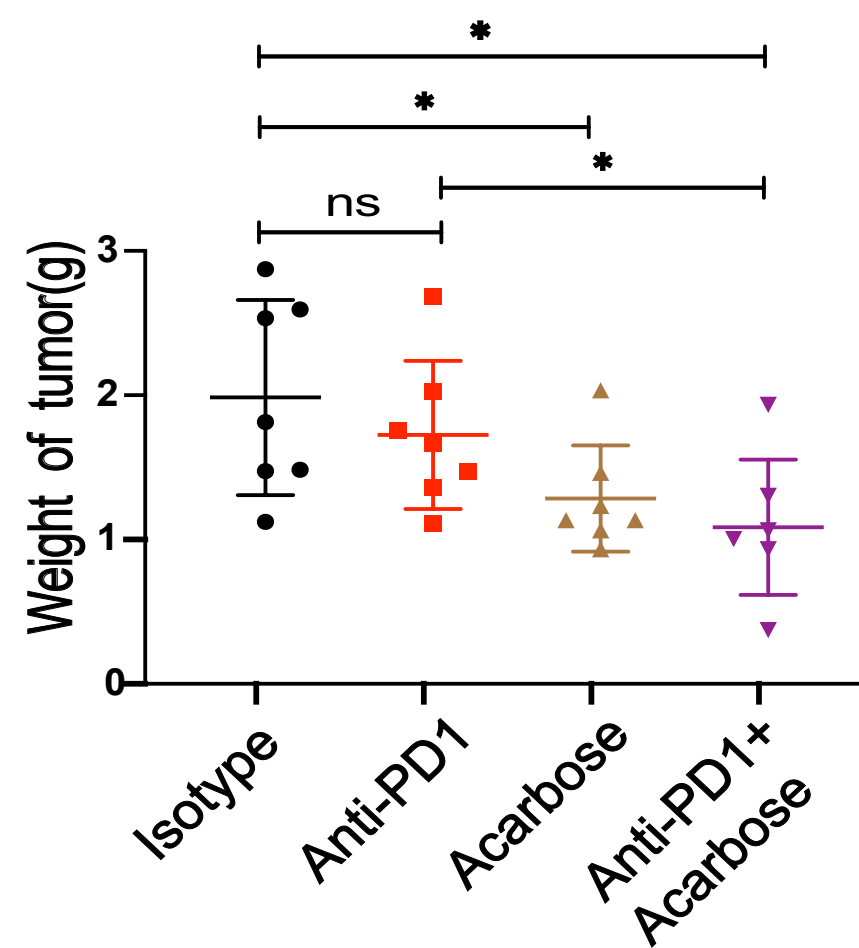

D

B16F10 tumor in C57BL/6 mice

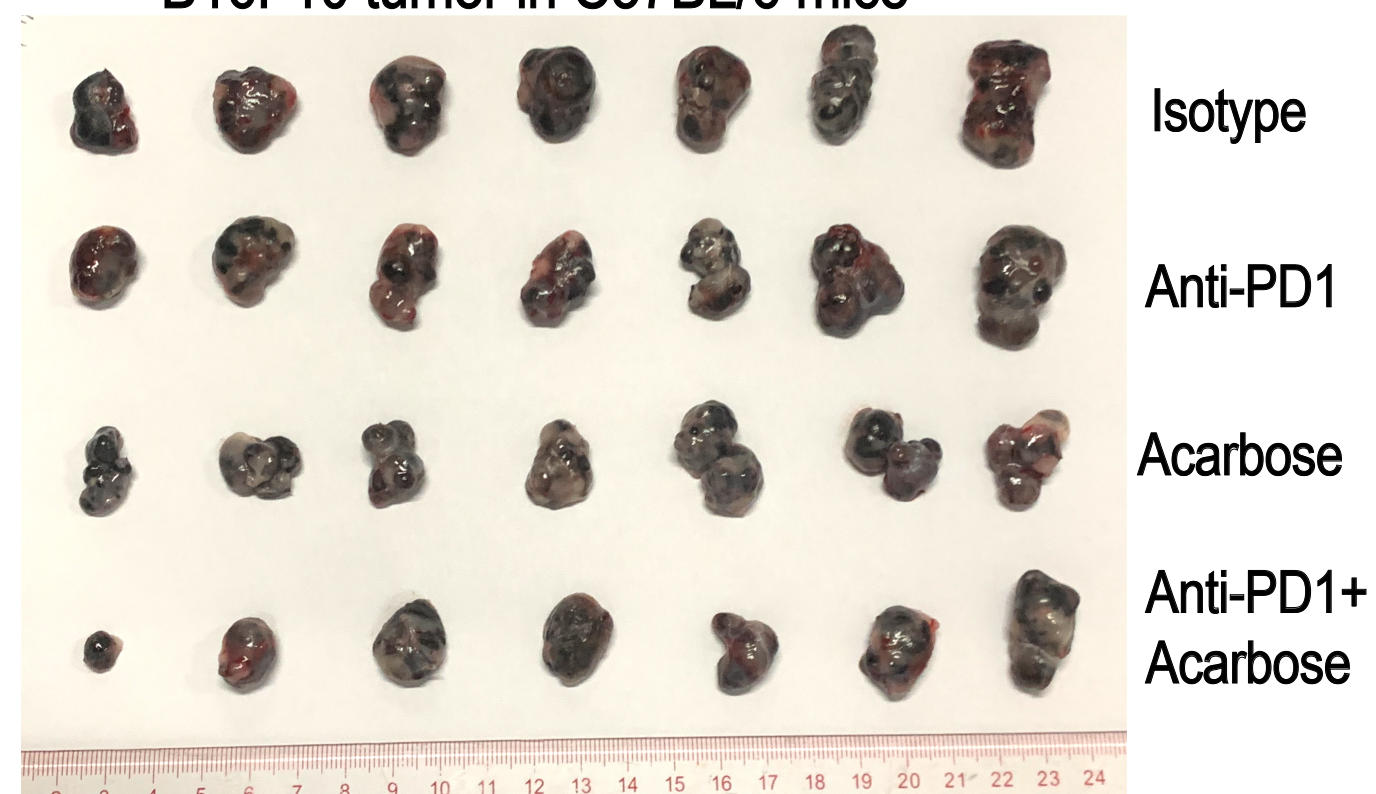

E

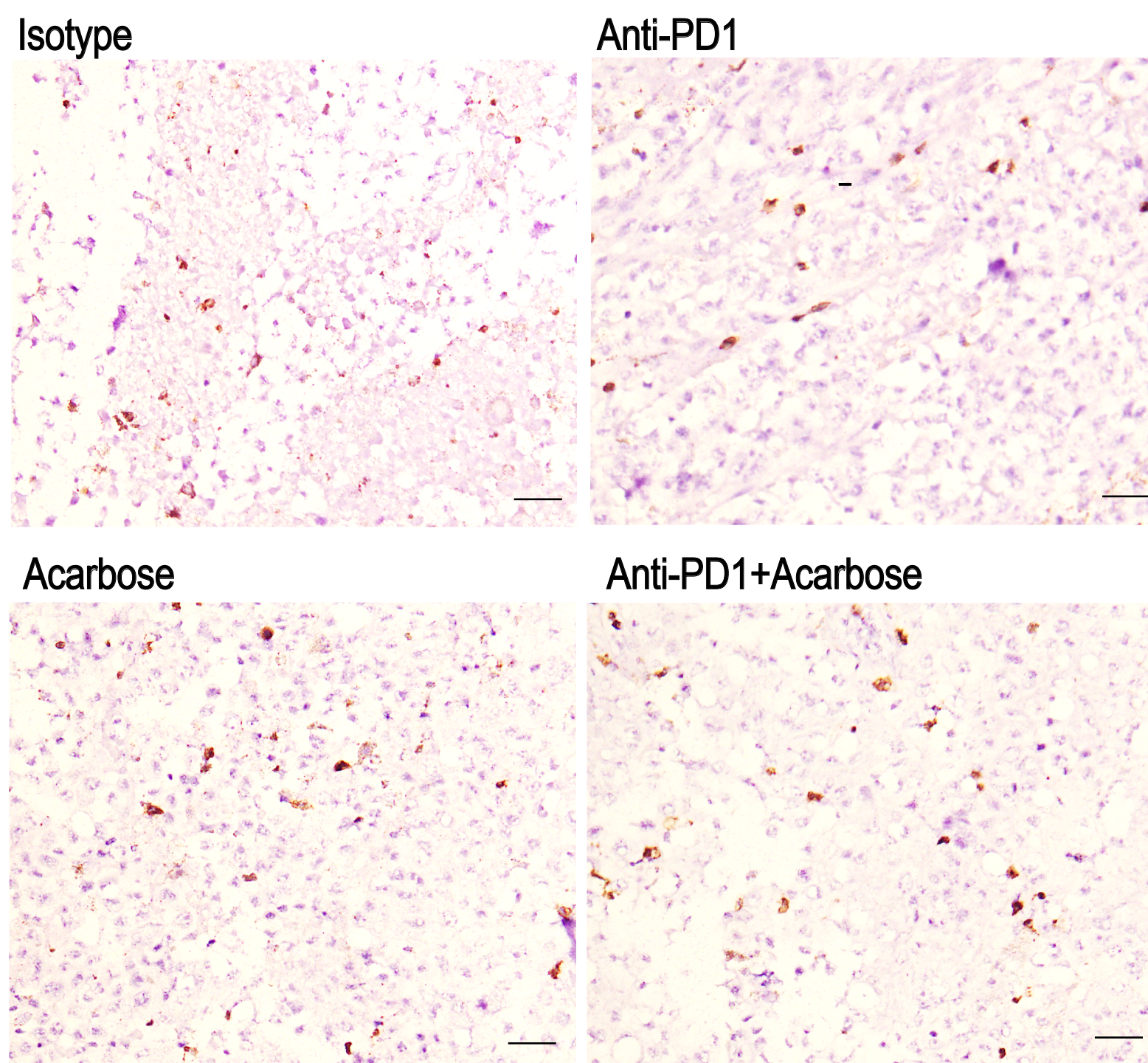

F

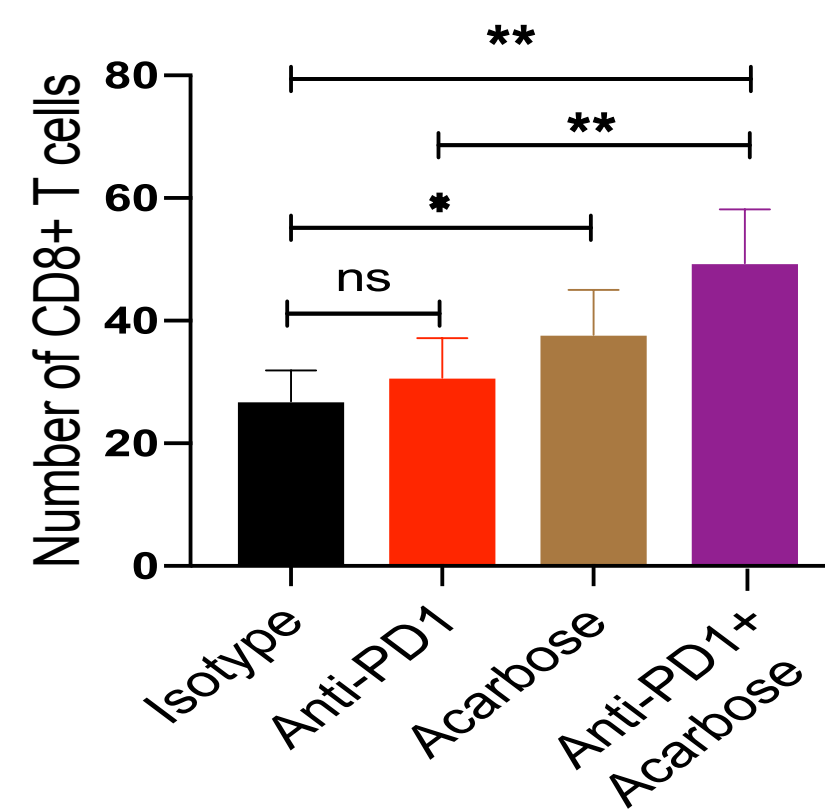

B16F10 tumor in C57BL/6 mice

Supplement: Supplementary Materials — Fig. S1: acarbose inhibits melanoma tumor growth and enhances tumor immune responses to anti-PD1. Figure S2: sitagliptin has no effects on melanoma tumor growth and tumor immune responses to anti-PD1. Figure S3: metformin has no effects on melanoma tumor growth and tumor immune responses to anti-PD1. Figure S4: glimepiride enhances melanoma tumor immune responses to anti-PD1. Figure S5: pioglitazone inhibits melanoma tumor growth, but anti-PD1 weakens tumor inhibition of pioglitazone. Figure S6: insulin has no effects on melanoma tumor growth and tumor immune responses to anti-PD1. Figure S7: compare the effect of the six antidiabetic drugs on MC38 tumor inhibition. Figure S8: compare the effect of the six antidiabetic drugs on CT26 tumor inhibition. Figure S9: compare the effect of the six antidiabetic drugs on B16F10 tumor inhibition. Figure S10: the expression of IGF1R, IGF2R, and PPARG was negatively correlated with the number of infiltrated CD8+ T cells in colorectal cancer. Figure S11: the inhibitory effect of each antidiabetic drugs on CT26 cell proliferation. Figure S12: the effect of acarbose and insulin on anti-PD1 tumor inhibition was not related to blood glucose. Figure S13: the mice weight of each group in the day of MC38 tumor harvested. Table.S1: the weight of tumor after different Intervention [file 2651790.f1.zip › Figure S1.pdf]

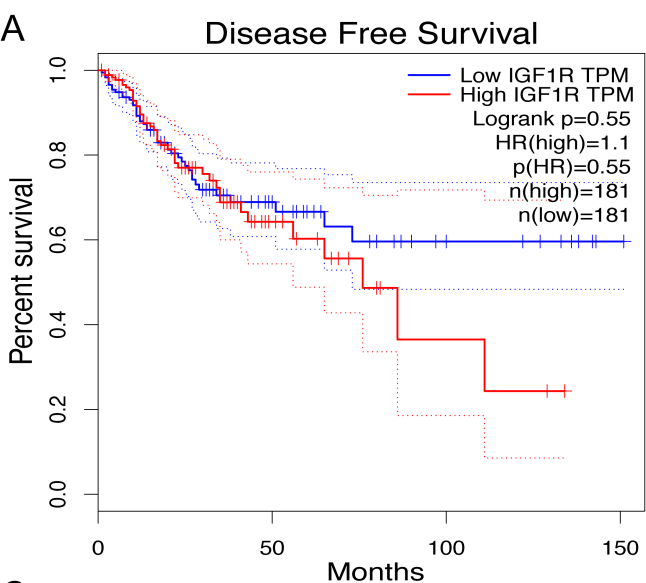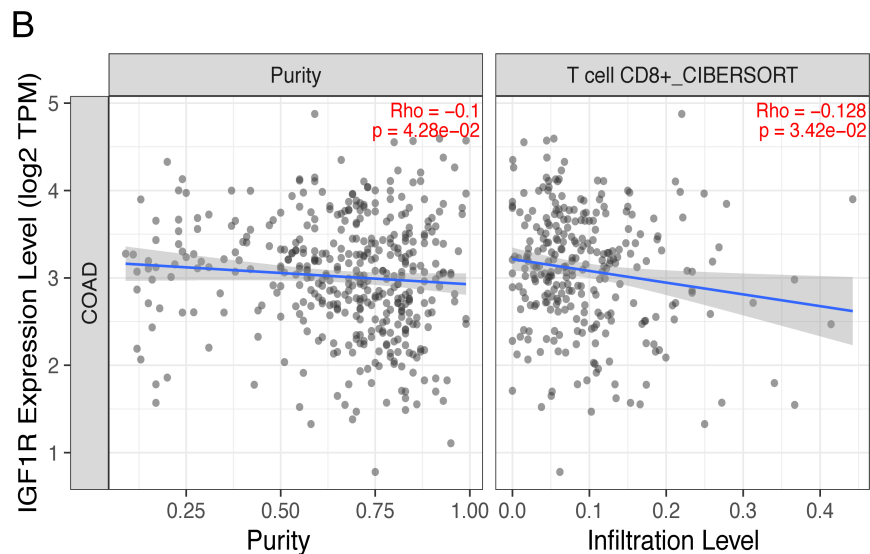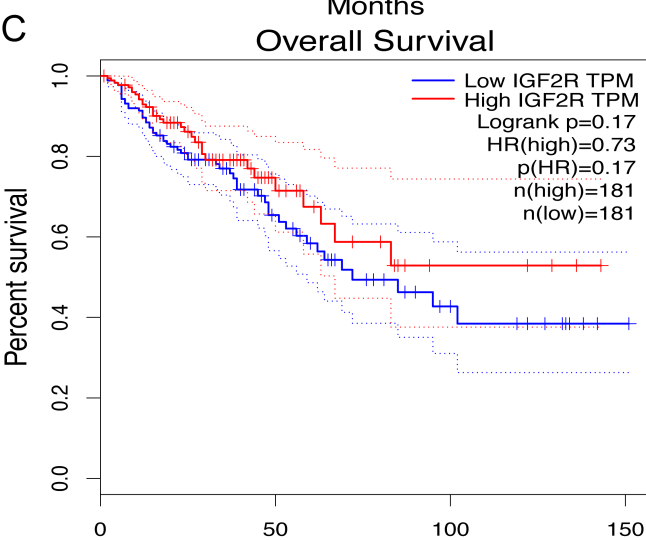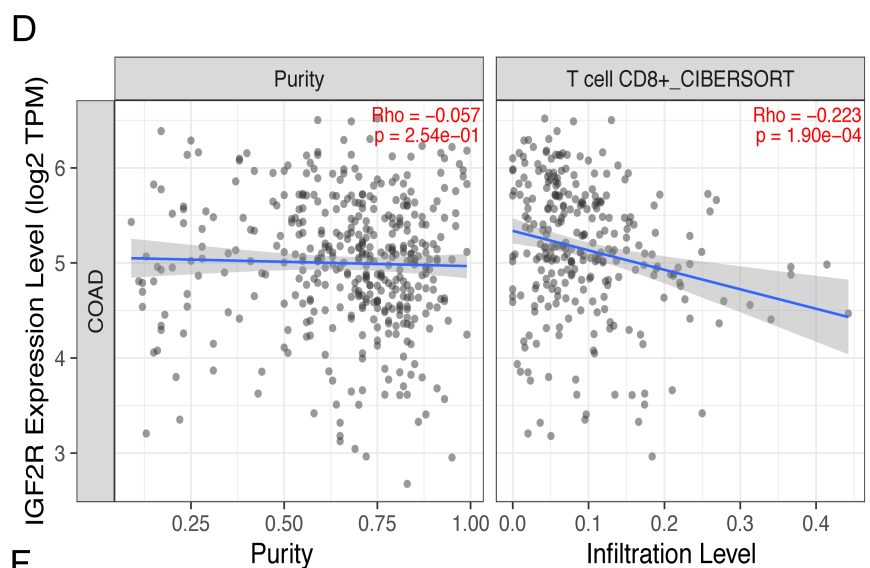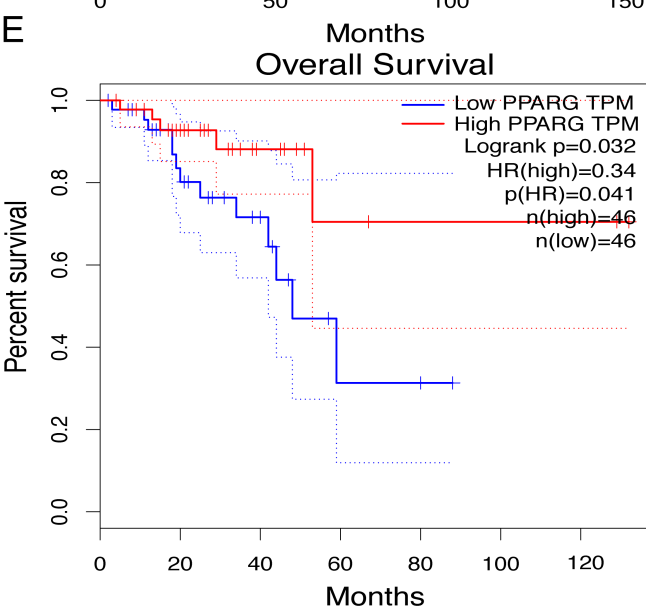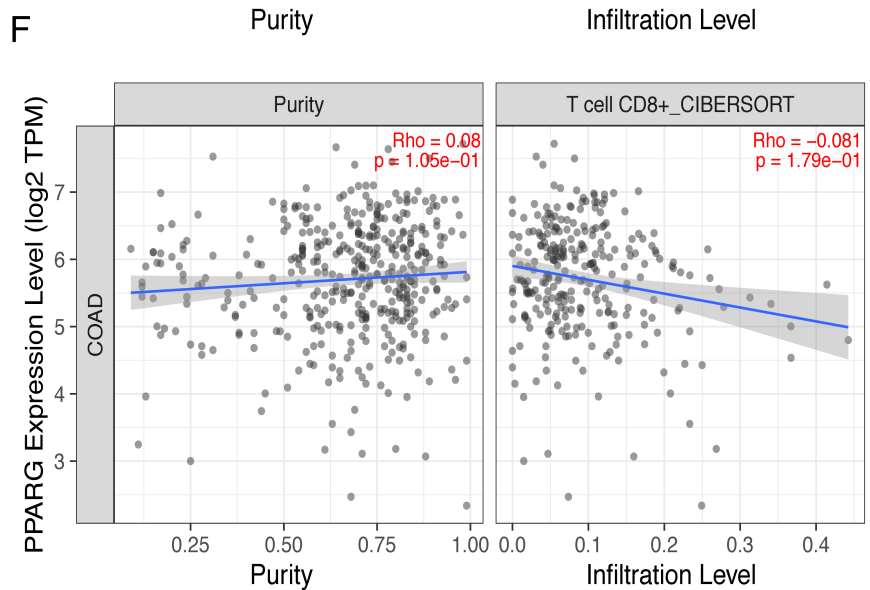

Supplement: Supplementary Materials — Fig. S1: acarbose inhibits melanoma tumor growth and enhances tumor immune responses to anti-PD1. Figure S2: sitagliptin has no effects on melanoma tumor growth and tumor immune responses to anti-PD1. Figure S3: metformin has no effects on melanoma tumor growth and tumor immune responses to anti-PD1. Figure S4: glimepiride enhances melanoma tumor immune responses to anti-PD1. Figure S5: pioglitazone inhibits melanoma tumor growth, but anti-PD1 weakens tumor inhibition of pioglitazone. Figure S6: insulin has no effects on melanoma tumor growth and tumor immune responses to anti-PD1. Figure S7: compare the effect of the six antidiabetic drugs on MC38 tumor inhibition. Figure S8: compare the effect of the six antidiabetic drugs on CT26 tumor inhibition. Figure S9: compare the effect of the six antidiabetic drugs on B16F10 tumor inhibition. Figure S10: the expression of IGF1R, IGF2R, and PPARG was negatively correlated with the number of infiltrated CD8+ T cells in colorectal cancer. Figure S11: the inhibitory effect of each antidiabetic drugs on CT26 cell proliferation. Figure S12: the effect of acarbose and insulin on anti-PD1 tumor inhibition was not related to blood glucose. Figure S13: the mice weight of each group in the day of MC38 tumor harvested. Table.S1: the weight of tumor after different Intervention [file 2651790.f1.zip › Figure S10.pdf]

# CT26

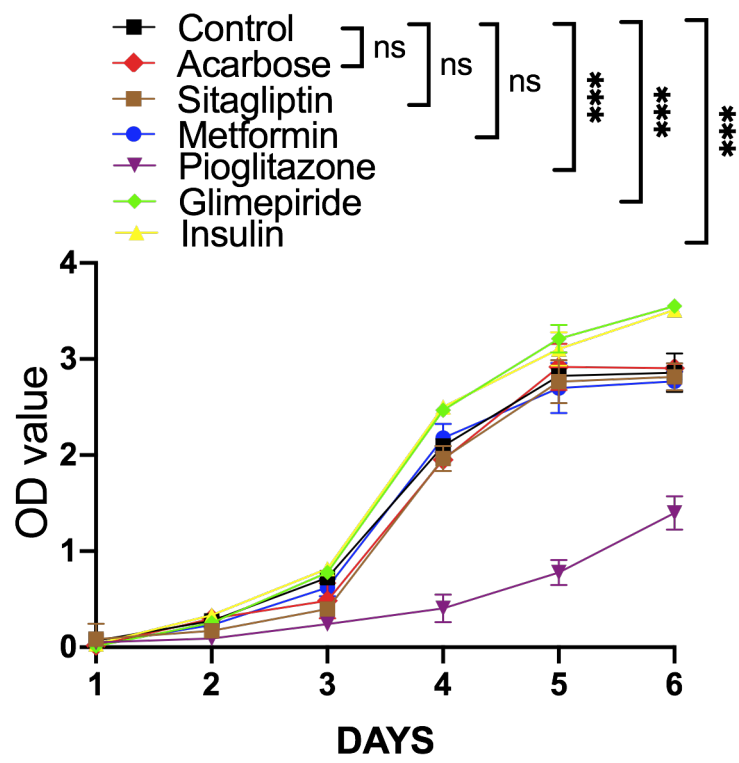

Supplement: Supplementary Materials — Fig. S1: acarbose inhibits melanoma tumor growth and enhances tumor immune responses to anti-PD1. Figure S2: sitagliptin has no effects on melanoma tumor growth and tumor immune responses to anti-PD1. Figure S3: metformin has no effects on melanoma tumor growth and tumor immune responses to anti-PD1. Figure S4: glimepiride enhances melanoma tumor immune responses to anti-PD1. Figure S5: pioglitazone inhibits melanoma tumor growth, but anti-PD1 weakens tumor inhibition of pioglitazone. Figure S6: insulin has no effects on melanoma tumor growth and tumor immune responses to anti-PD1. Figure S7: compare the effect of the six antidiabetic drugs on MC38 tumor inhibition. Figure S8: compare the effect of the six antidiabetic drugs on CT26 tumor inhibition. Figure S9: compare the effect of the six antidiabetic drugs on B16F10 tumor inhibition. Figure S10: the expression of IGF1R, IGF2R, and PPARG was negatively correlated with the number of infiltrated CD8+ T cells in colorectal cancer. Figure S11: the inhibitory effect of each antidiabetic drugs on CT26 cell proliferation. Figure S12: the effect of acarbose and insulin on anti-PD1 tumor inhibition was not related to blood glucose. Figure S13: the mice weight of each group in the day of MC38 tumor harvested. Table.S1: the weight of tumor after different Intervention [file 2651790.f1.zip › Figure S11.pdf]

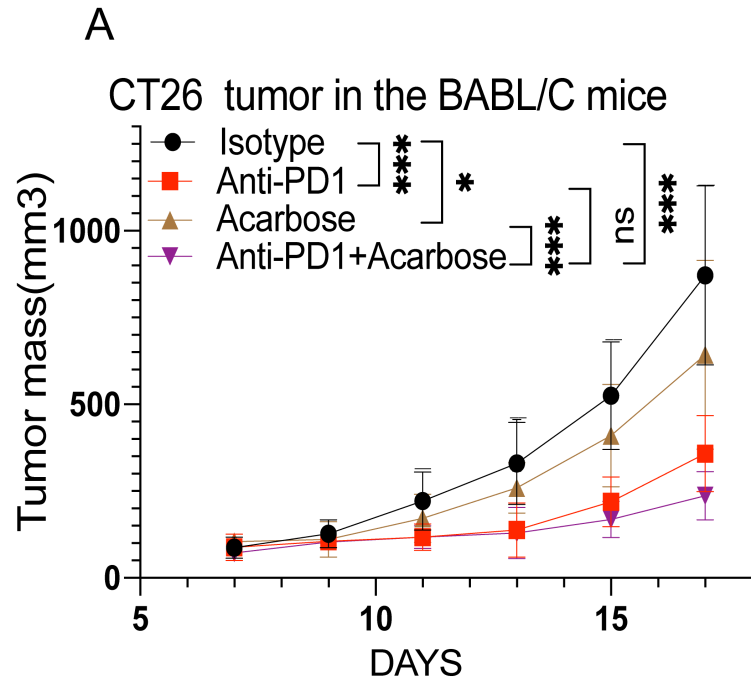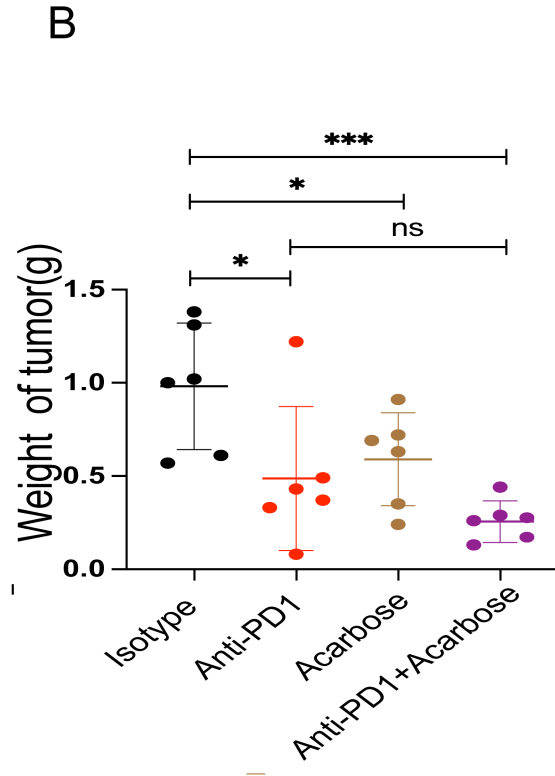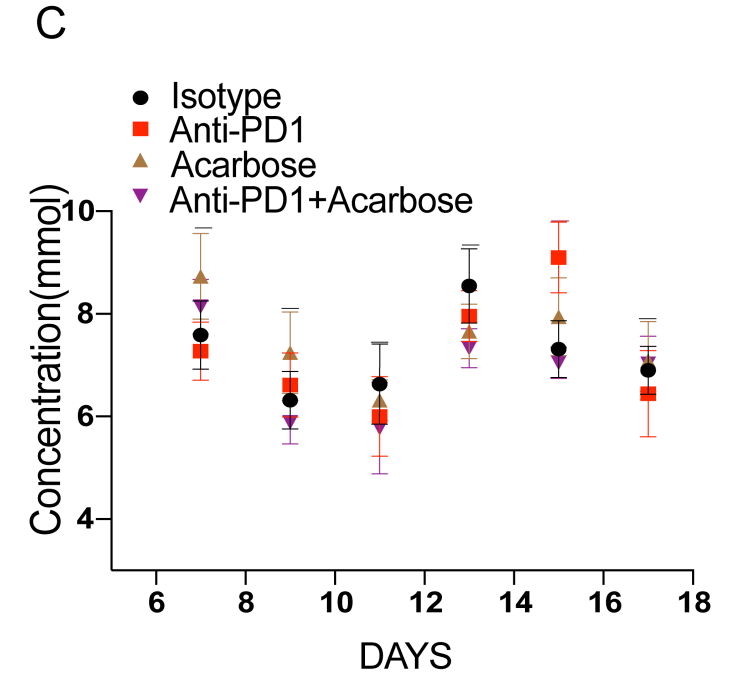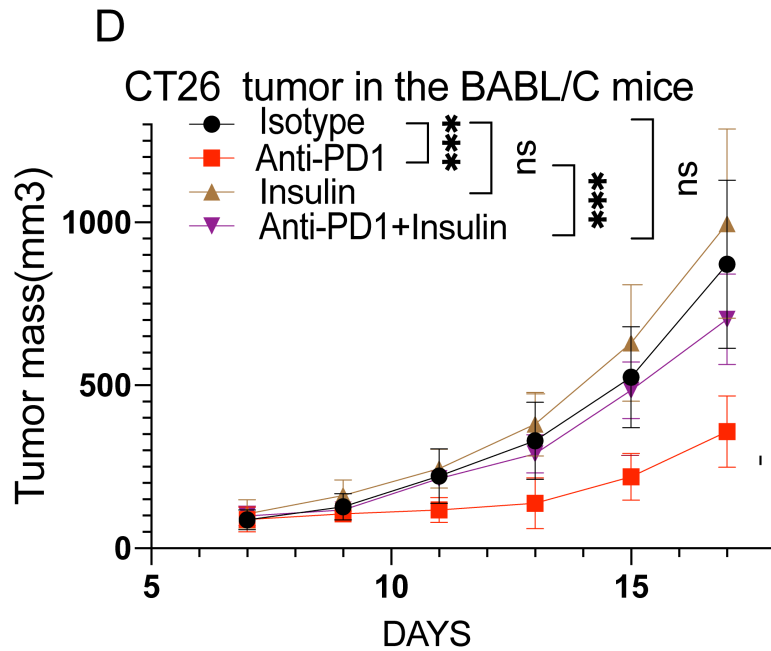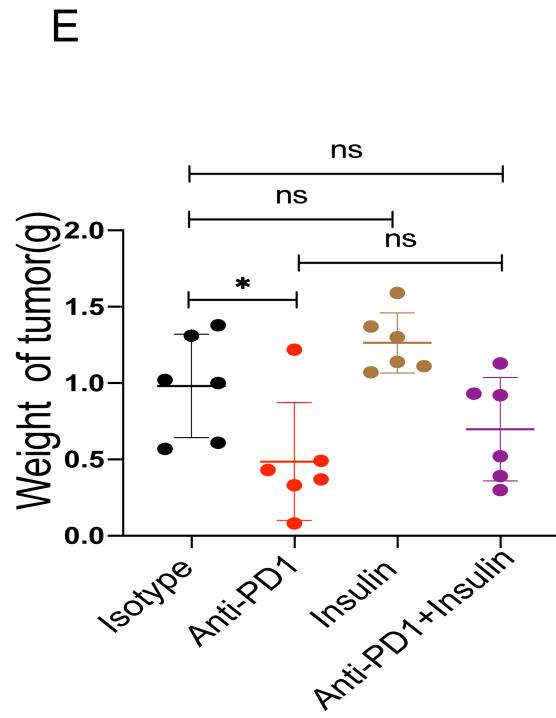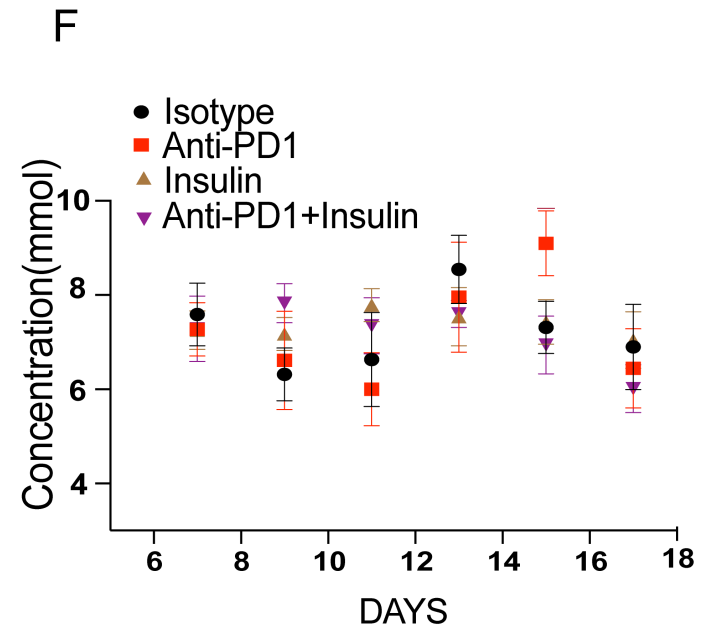

Supplement: Supplementary Materials — Fig. S1: acarbose inhibits melanoma tumor growth and enhances tumor immune responses to anti-PD1. Figure S2: sitagliptin has no effects on melanoma tumor growth and tumor immune responses to anti-PD1. Figure S3: metformin has no effects on melanoma tumor growth and tumor immune responses to anti-PD1. Figure S4: glimepiride enhances melanoma tumor immune responses to anti-PD1. Figure S5: pioglitazone inhibits melanoma tumor growth, but anti-PD1 weakens tumor inhibition of pioglitazone. Figure S6: insulin has no effects on melanoma tumor growth and tumor immune responses to anti-PD1. Figure S7: compare the effect of the six antidiabetic drugs on MC38 tumor inhibition. Figure S8: compare the effect of the six antidiabetic drugs on CT26 tumor inhibition. Figure S9: compare the effect of the six antidiabetic drugs on B16F10 tumor inhibition. Figure S10: the expression of IGF1R, IGF2R, and PPARG was negatively correlated with the number of infiltrated CD8+ T cells in colorectal cancer. Figure S11: the inhibitory effect of each antidiabetic drugs on CT26 cell proliferation. Figure S12: the effect of acarbose and insulin on anti-PD1 tumor inhibition was not related to blood glucose. Figure S13: the mice weight of each group in the day of MC38 tumor harvested. Table.S1: the weight of tumor after different Intervention [file 2651790.f1.zip › Figure S12.pdf]

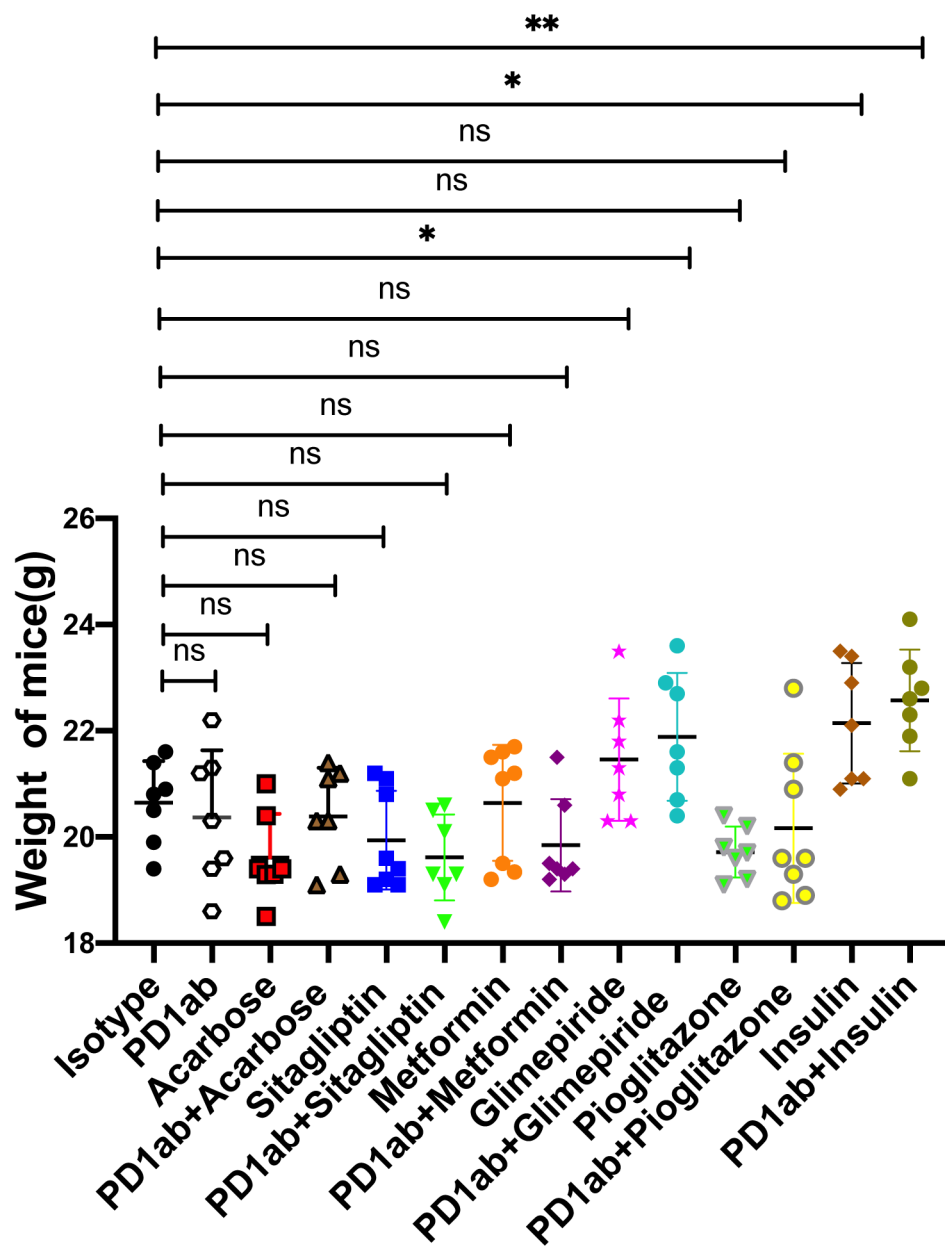

Supplement: Supplementary Materials — Fig. S1: acarbose inhibits melanoma tumor growth and enhances tumor immune responses to anti-PD1. Figure S2: sitagliptin has no effects on melanoma tumor growth and tumor immune responses to anti-PD1. Figure S3: metformin has no effects on melanoma tumor growth and tumor immune responses to anti-PD1. Figure S4: glimepiride enhances melanoma tumor immune responses to anti-PD1. Figure S5: pioglitazone inhibits melanoma tumor growth, but anti-PD1 weakens tumor inhibition of pioglitazone. Figure S6: insulin has no effects on melanoma tumor growth and tumor immune responses to anti-PD1. Figure S7: compare the effect of the six antidiabetic drugs on MC38 tumor inhibition. Figure S8: compare the effect of the six antidiabetic drugs on CT26 tumor inhibition. Figure S9: compare the effect of the six antidiabetic drugs on B16F10 tumor inhibition. Figure S10: the expression of IGF1R, IGF2R, and PPARG was negatively correlated with the number of infiltrated CD8+ T cells in colorectal cancer. Figure S11: the inhibitory effect of each antidiabetic drugs on CT26 cell proliferation. Figure S12: the effect of acarbose and insulin on anti-PD1 tumor inhibition was not related to blood glucose. Figure S13: the mice weight of each group in the day of MC38 tumor harvested. Table.S1: the weight of tumor after different Intervention [file 2651790.f1.zip › Figure S13.pdf]

B16F10 tumor in C57BL/6 mice

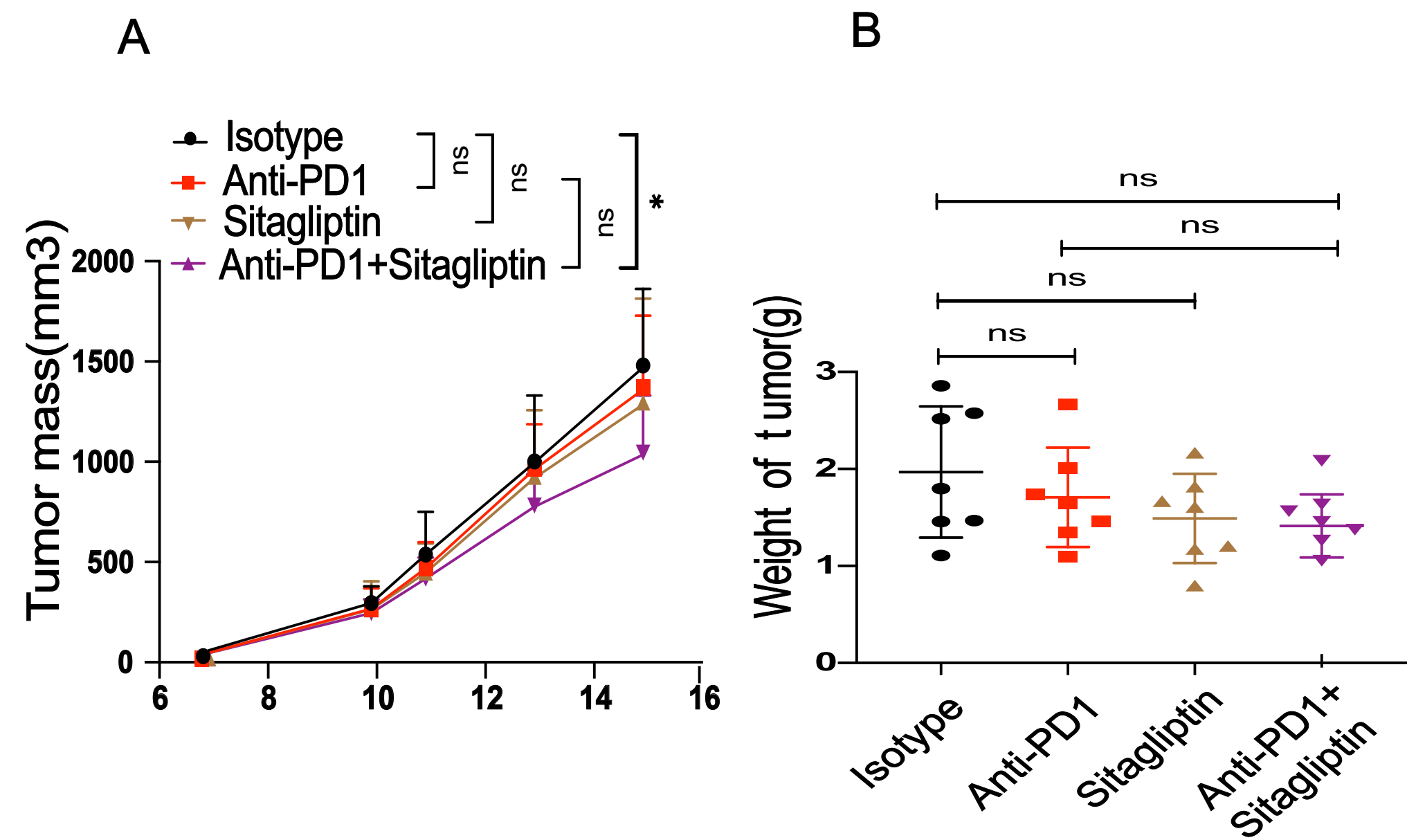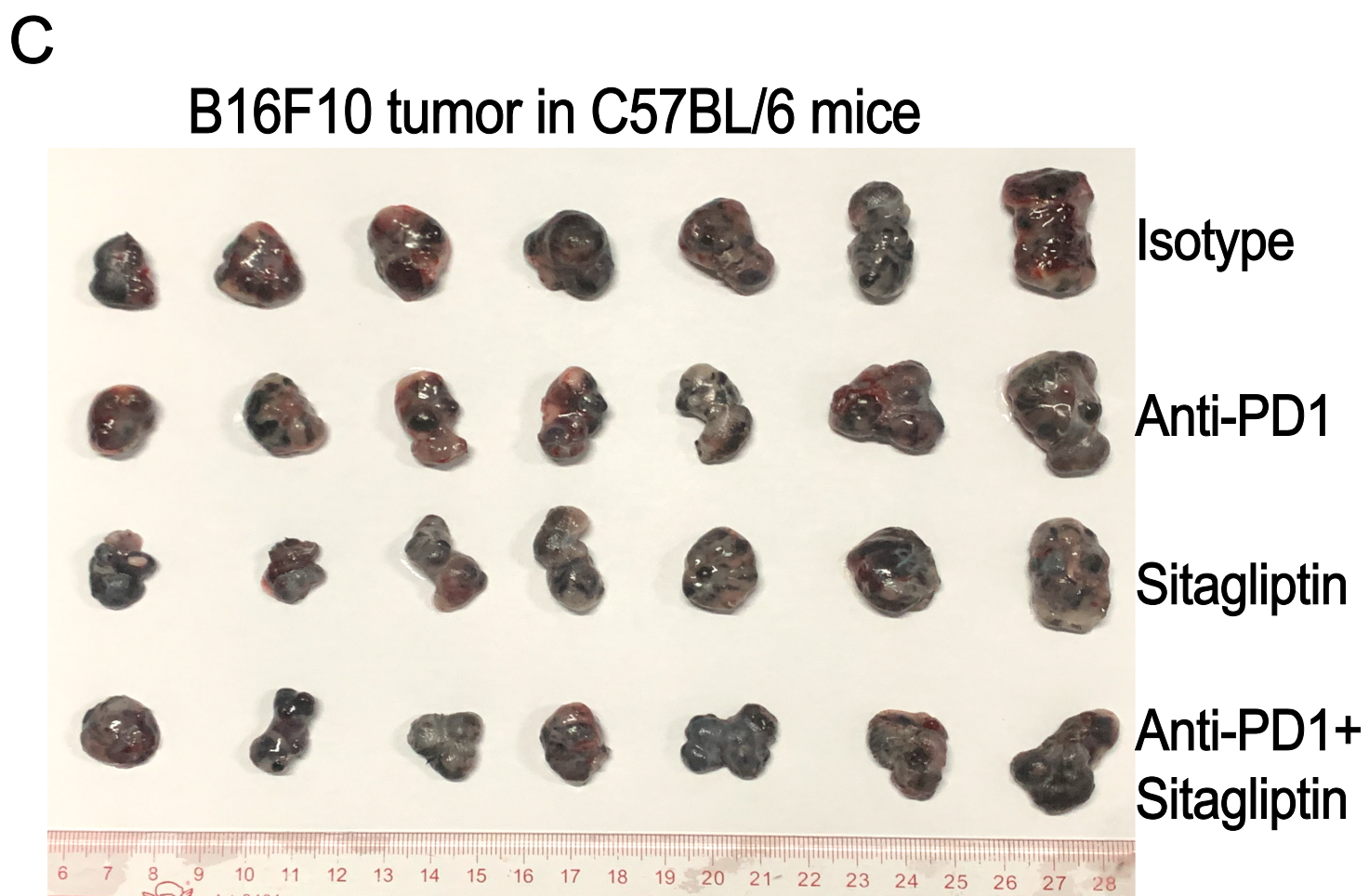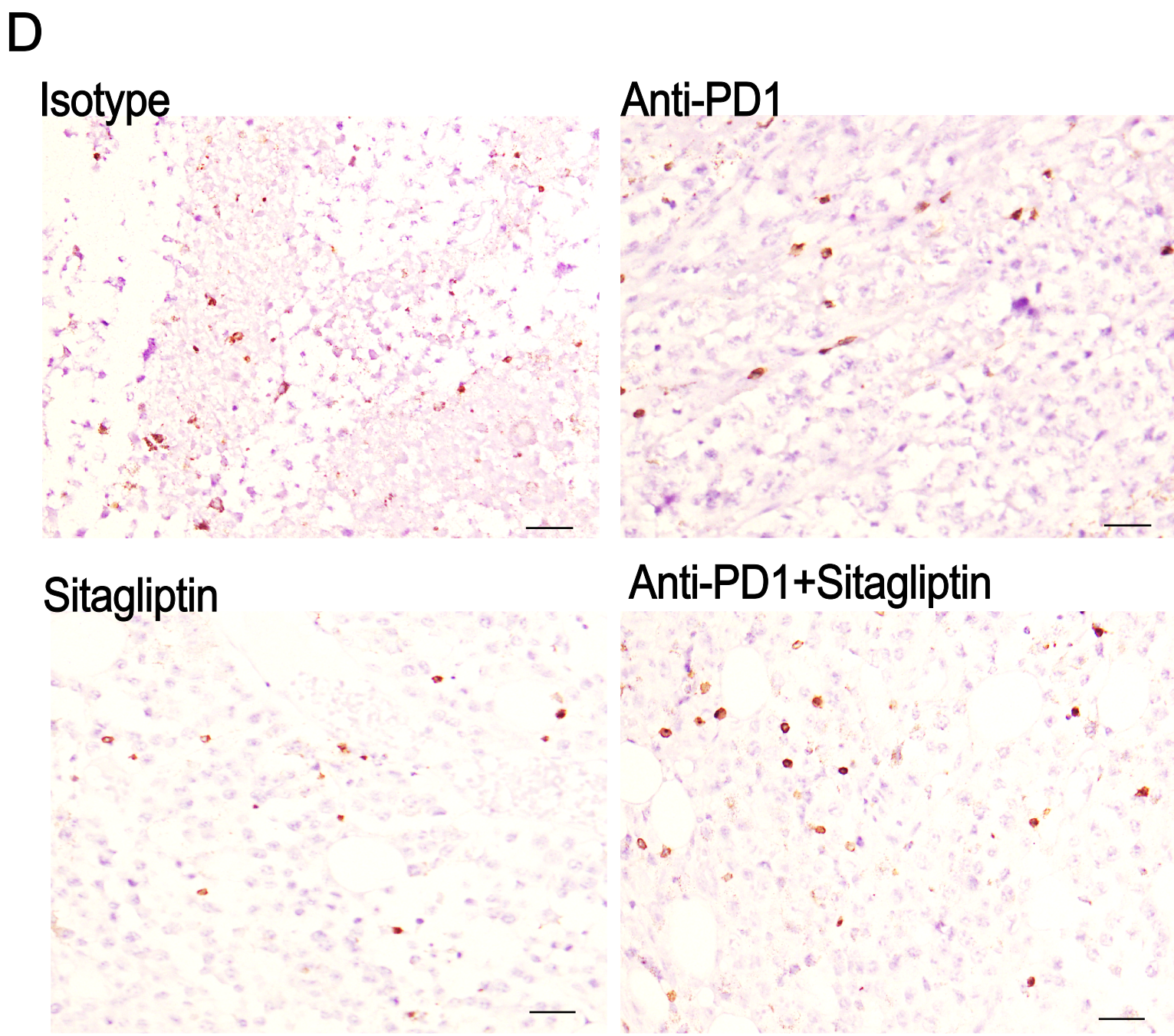

B16F10 tumor in C57BL/6 mice

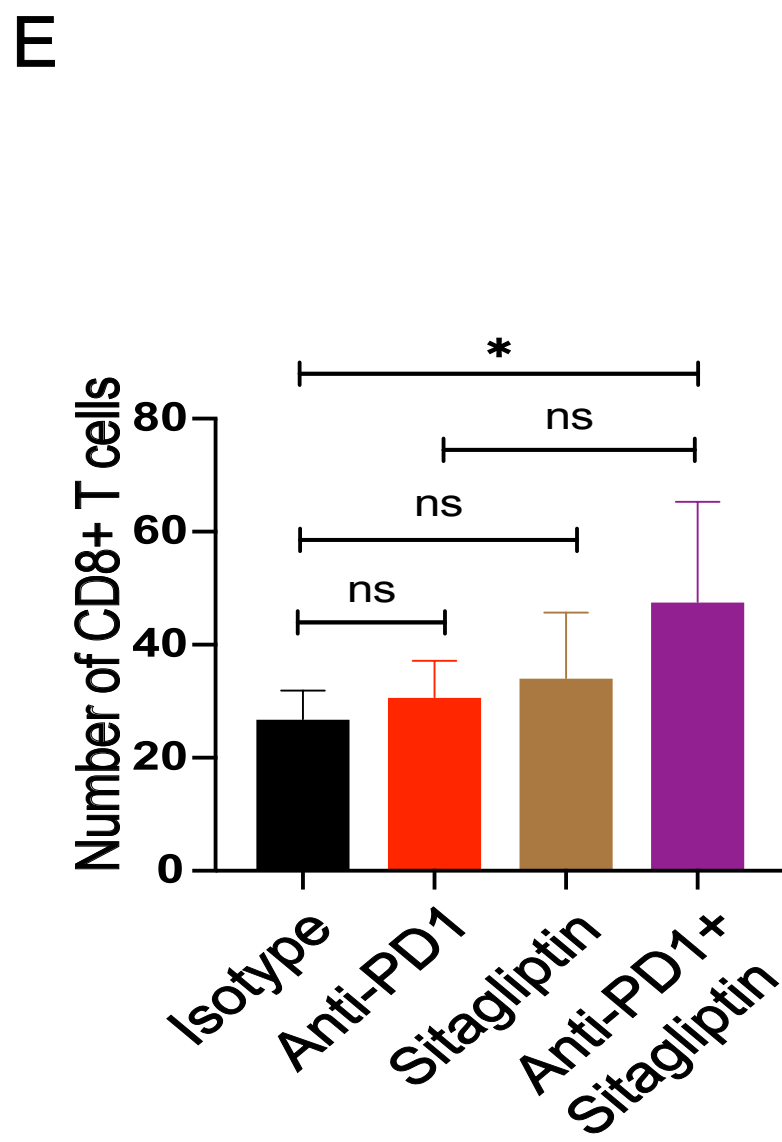

Supplement: Supplementary Materials — Fig. S1: acarbose inhibits melanoma tumor growth and enhances tumor immune responses to anti-PD1. Figure S2: sitagliptin has no effects on melanoma tumor growth and tumor immune responses to anti-PD1. Figure S3: metformin has no effects on melanoma tumor growth and tumor immune responses to anti-PD1. Figure S4: glimepiride enhances melanoma tumor immune responses to anti-PD1. Figure S5: pioglitazone inhibits melanoma tumor growth, but anti-PD1 weakens tumor inhibition of pioglitazone. Figure S6: insulin has no effects on melanoma tumor growth and tumor immune responses to anti-PD1. Figure S7: compare the effect of the six antidiabetic drugs on MC38 tumor inhibition. Figure S8: compare the effect of the six antidiabetic drugs on CT26 tumor inhibition. Figure S9: compare the effect of the six antidiabetic drugs on B16F10 tumor inhibition. Figure S10: the expression of IGF1R, IGF2R, and PPARG was negatively correlated with the number of infiltrated CD8+ T cells in colorectal cancer. Figure S11: the inhibitory effect of each antidiabetic drugs on CT26 cell proliferation. Figure S12: the effect of acarbose and insulin on anti-PD1 tumor inhibition was not related to blood glucose. Figure S13: the mice weight of each group in the day of MC38 tumor harvested. Table.S1: the weight of tumor after different Intervention [file 2651790.f1.zip › Figure S2.pdf]

B16F10 tumor in C57BL/6 mice

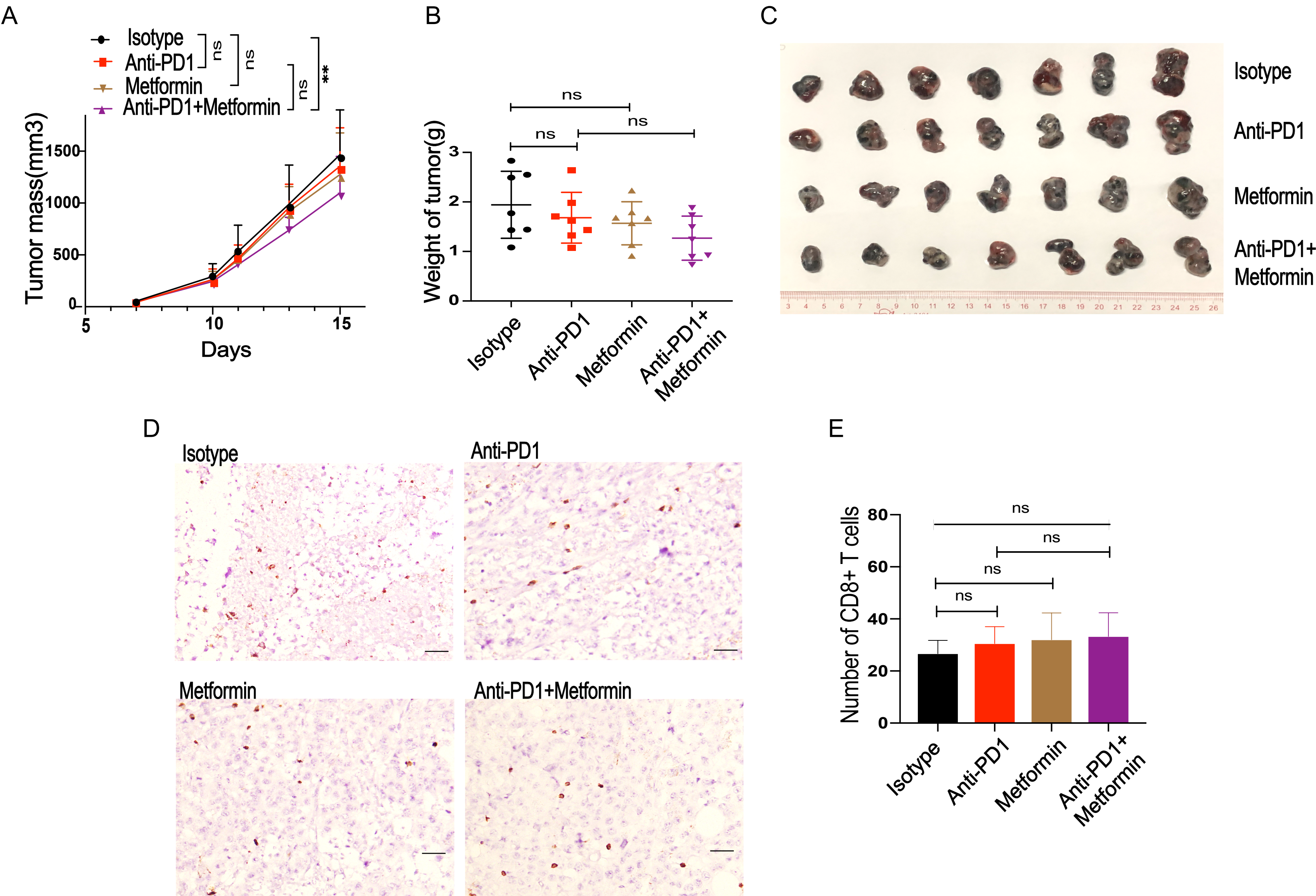

B16F10 tumor in C57BL/6 mice

Supplement: Supplementary Materials — Fig. S1: acarbose inhibits melanoma tumor growth and enhances tumor immune responses to anti-PD1. Figure S2: sitagliptin has no effects on melanoma tumor growth and tumor immune responses to anti-PD1. Figure S3: metformin has no effects on melanoma tumor growth and tumor immune responses to anti-PD1. Figure S4: glimepiride enhances melanoma tumor immune responses to anti-PD1. Figure S5: pioglitazone inhibits melanoma tumor growth, but anti-PD1 weakens tumor inhibition of pioglitazone. Figure S6: insulin has no effects on melanoma tumor growth and tumor immune responses to anti-PD1. Figure S7: compare the effect of the six antidiabetic drugs on MC38 tumor inhibition. Figure S8: compare the effect of the six antidiabetic drugs on CT26 tumor inhibition. Figure S9: compare the effect of the six antidiabetic drugs on B16F10 tumor inhibition. Figure S10: the expression of IGF1R, IGF2R, and PPARG was negatively correlated with the number of infiltrated CD8+ T cells in colorectal cancer. Figure S11: the inhibitory effect of each antidiabetic drugs on CT26 cell proliferation. Figure S12: the effect of acarbose and insulin on anti-PD1 tumor inhibition was not related to blood glucose. Figure S13: the mice weight of each group in the day of MC38 tumor harvested. Table.S1: the weight of tumor after different Intervention [file 2651790.f1.zip › Figure S3.pdf]

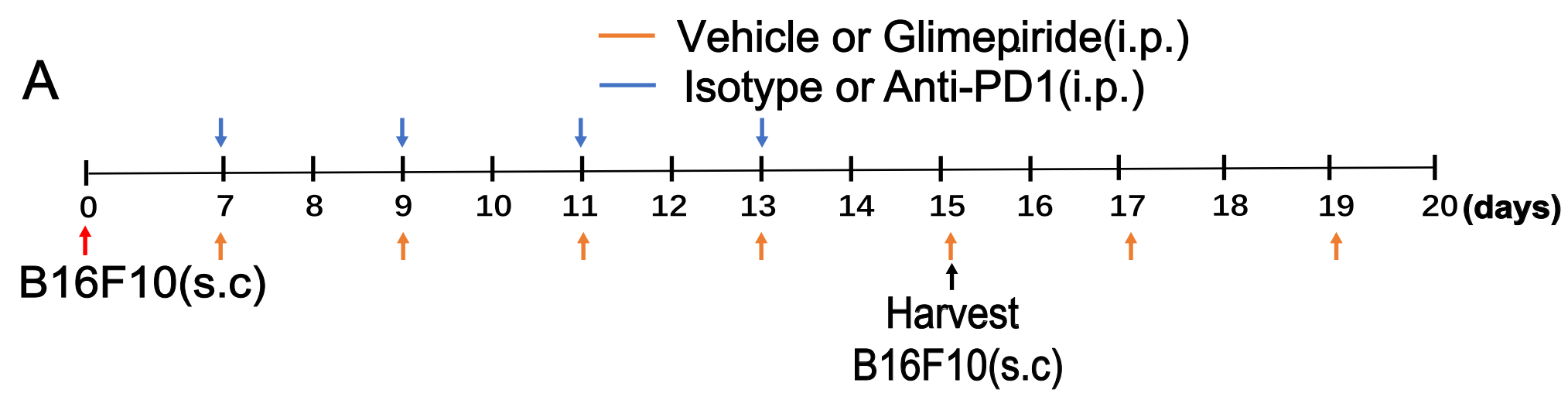

## B16F10 tumor in C57BL/6 mice

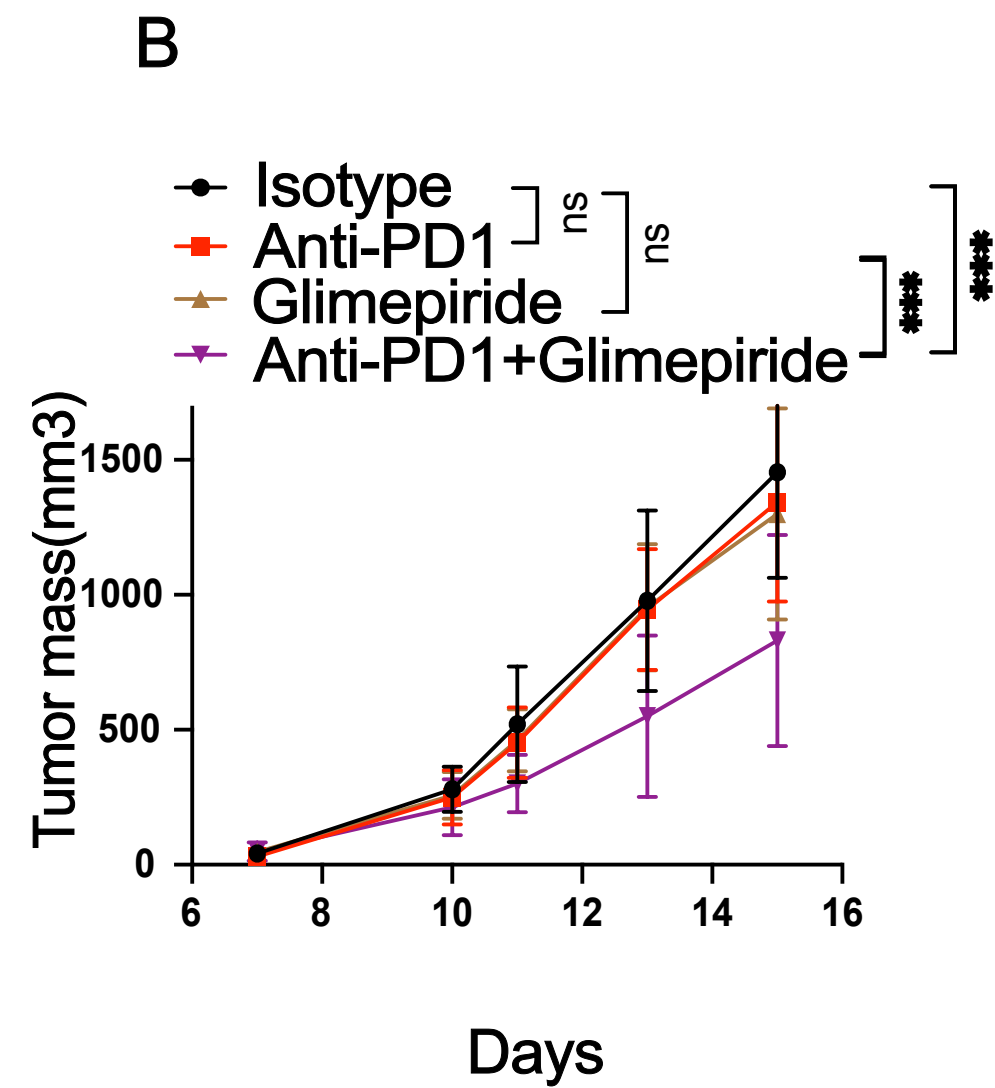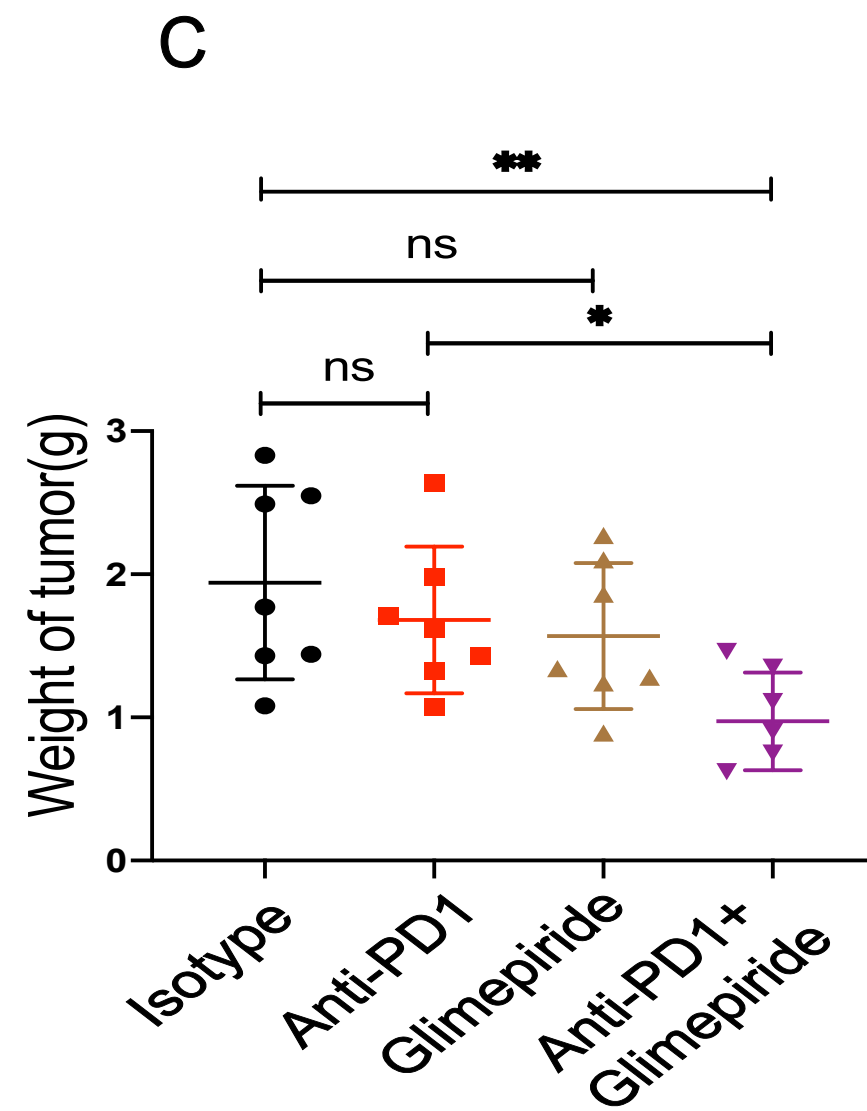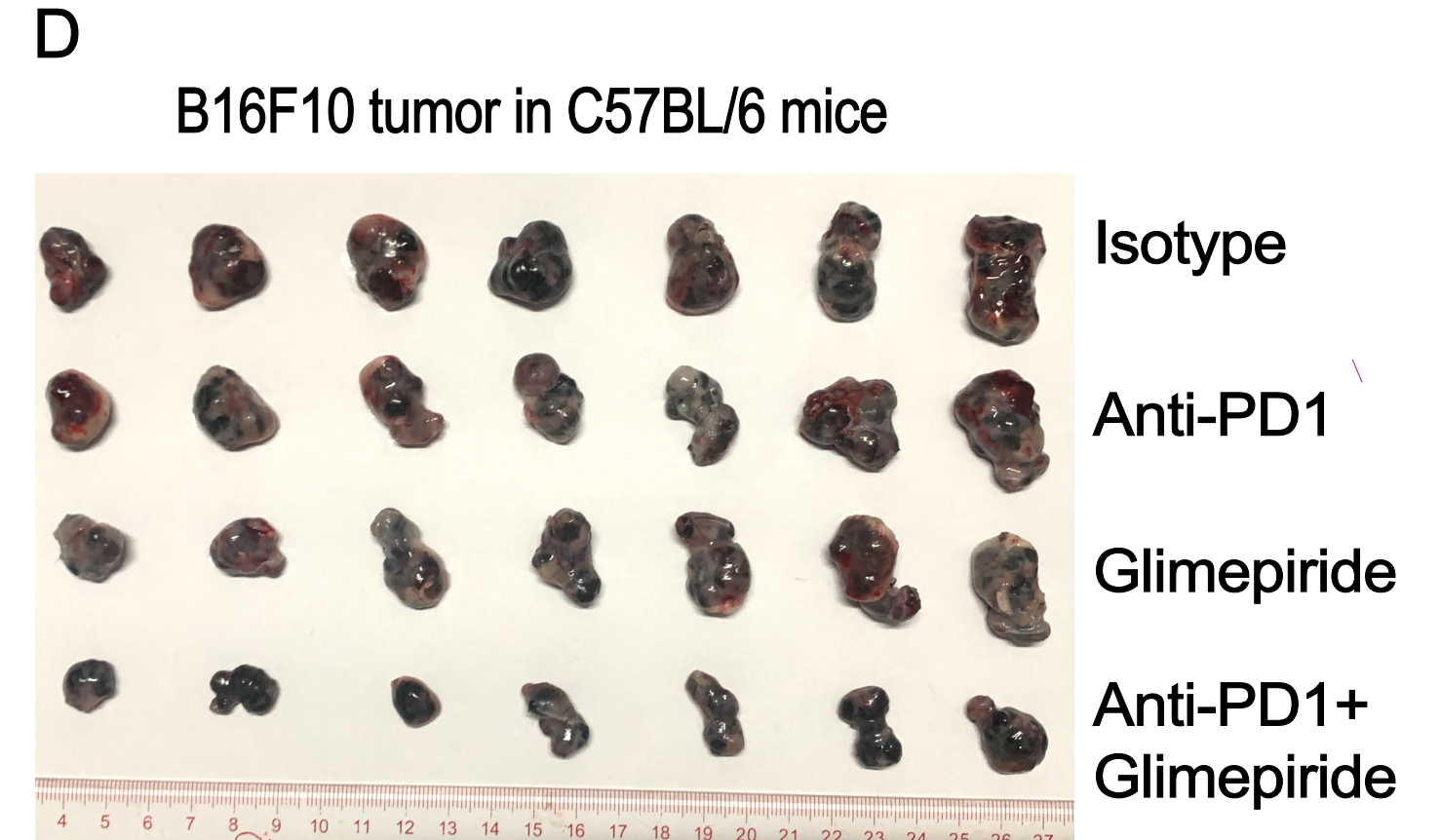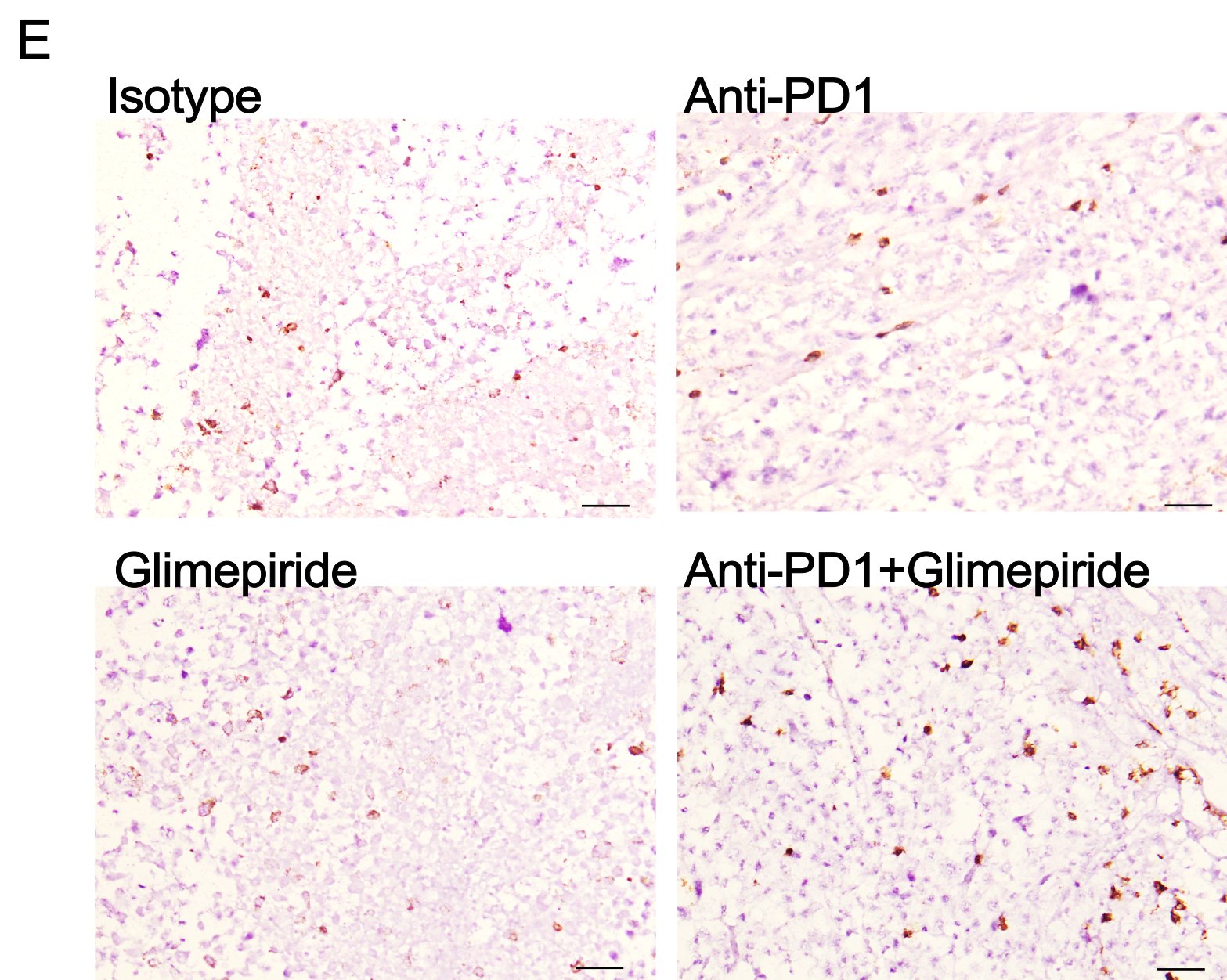

## B16F10 tumor in C57BL/6 mice

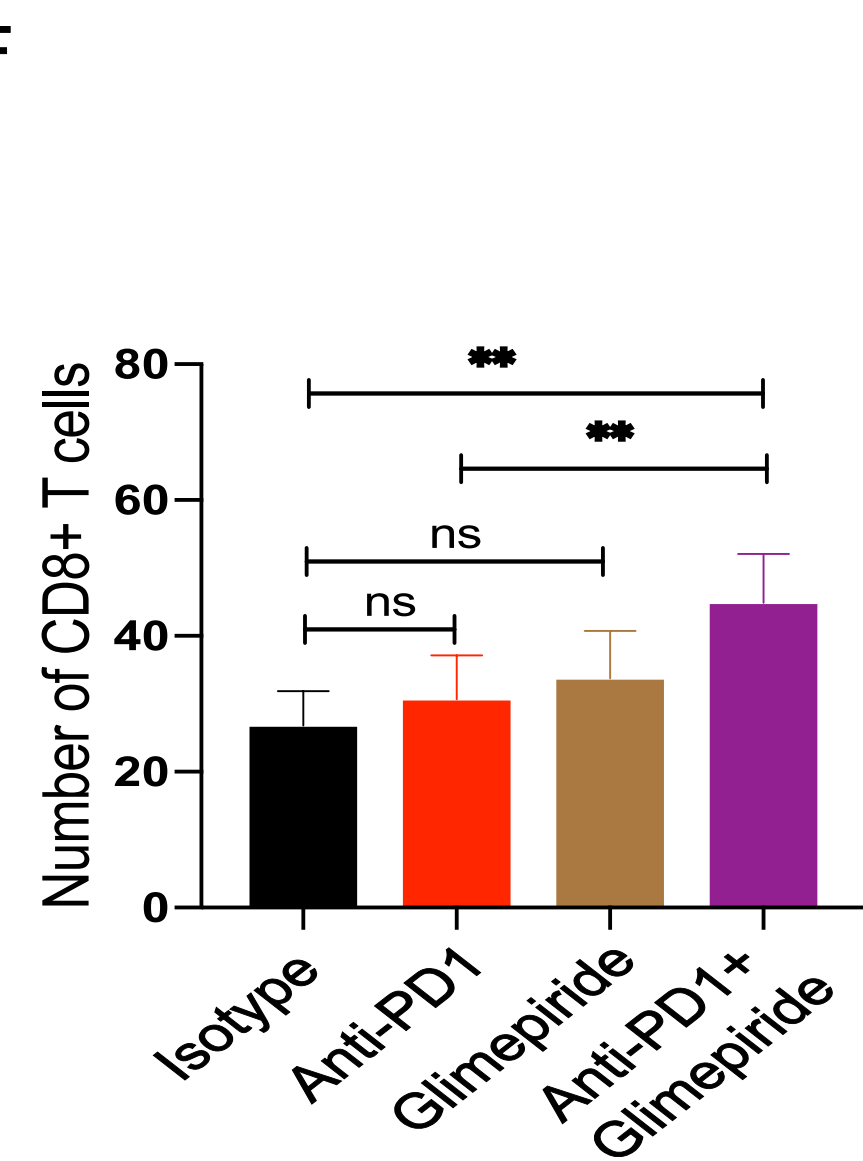

Supplement: Supplementary Materials — Fig. S1: acarbose inhibits melanoma tumor growth and enhances tumor immune responses to anti-PD1. Figure S2: sitagliptin has no effects on melanoma tumor growth and tumor immune responses to anti-PD1. Figure S3: metformin has no effects on melanoma tumor growth and tumor immune responses to anti-PD1. Figure S4: glimepiride enhances melanoma tumor immune responses to anti-PD1. Figure S5: pioglitazone inhibits melanoma tumor growth, but anti-PD1 weakens tumor inhibition of pioglitazone. Figure S6: insulin has no effects on melanoma tumor growth and tumor immune responses to anti-PD1. Figure S7: compare the effect of the six antidiabetic drugs on MC38 tumor inhibition. Figure S8: compare the effect of the six antidiabetic drugs on CT26 tumor inhibition. Figure S9: compare the effect of the six antidiabetic drugs on B16F10 tumor inhibition. Figure S10: the expression of IGF1R, IGF2R, and PPARG was negatively correlated with the number of infiltrated CD8+ T cells in colorectal cancer. Figure S11: the inhibitory effect of each antidiabetic drugs on CT26 cell proliferation. Figure S12: the effect of acarbose and insulin on anti-PD1 tumor inhibition was not related to blood glucose. Figure S13: the mice weight of each group in the day of MC38 tumor harvested. Table.S1: the weight of tumor after different Intervention [file 2651790.f1.zip › Figure S4.pdf]

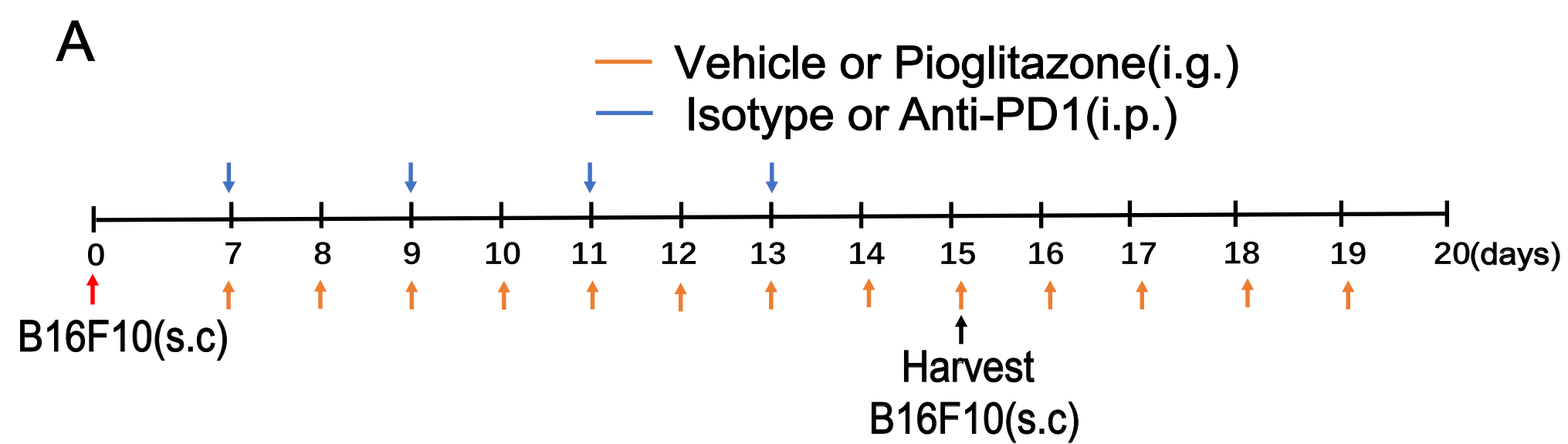

## B16F10 tumor in C57BL/6 mice

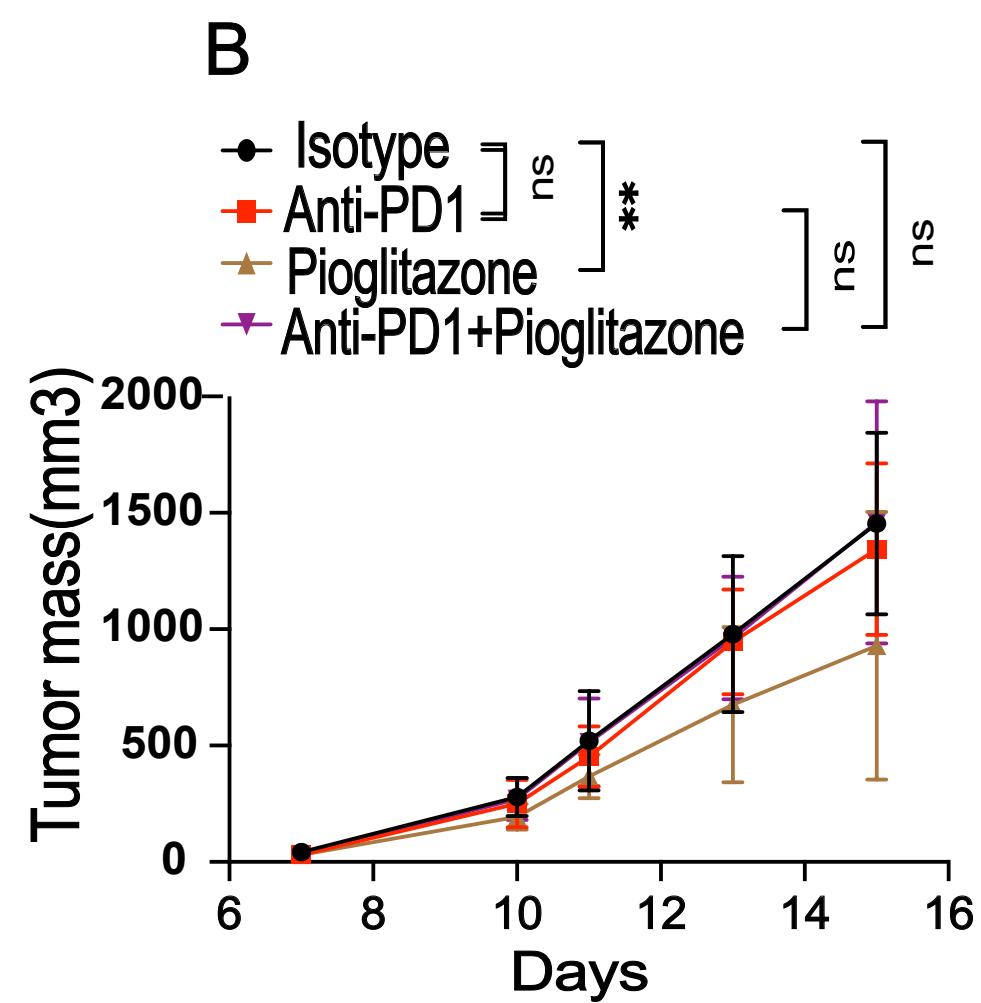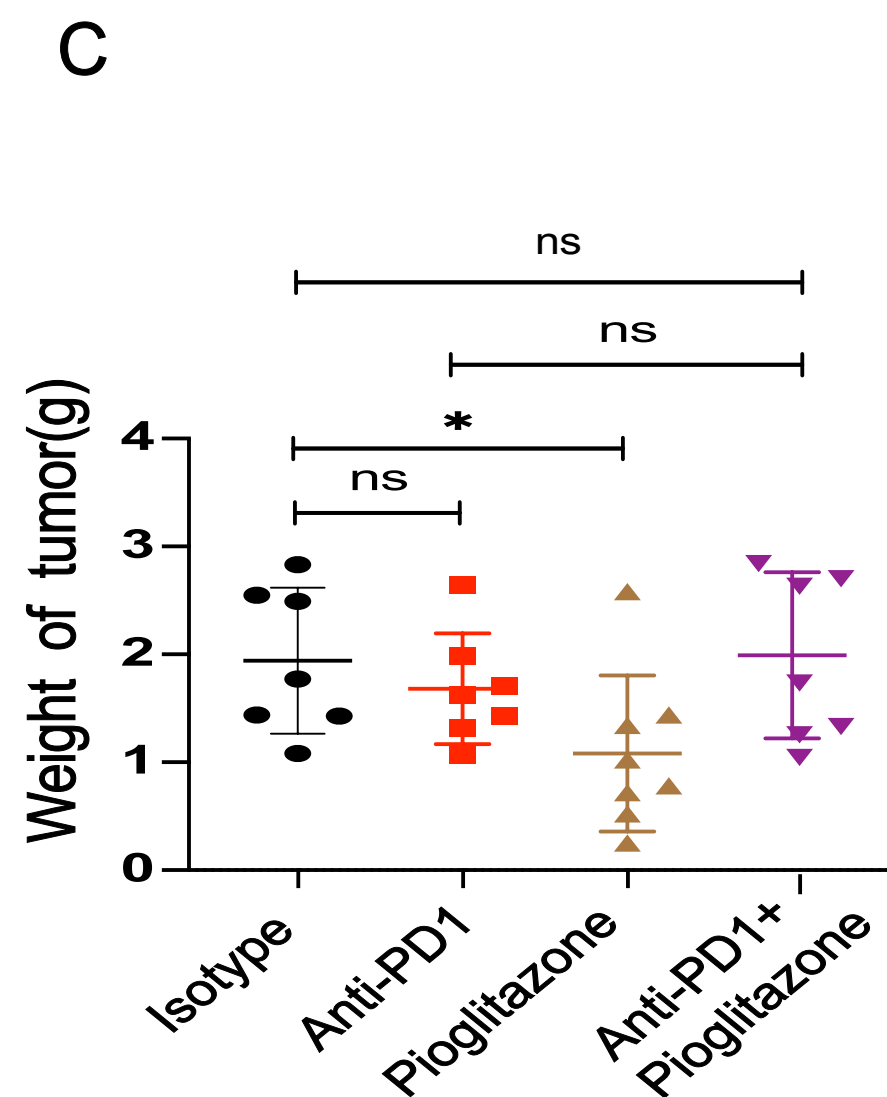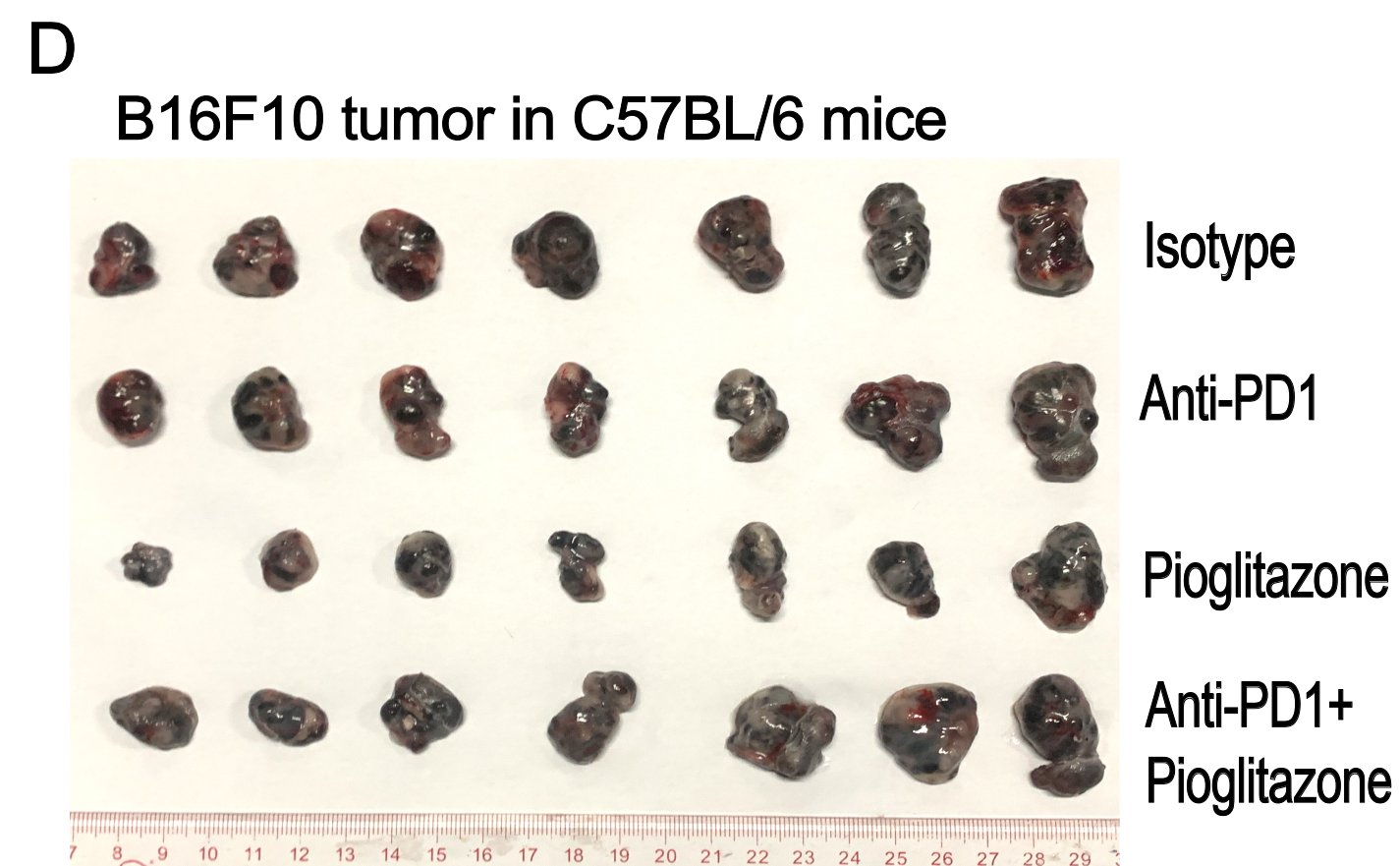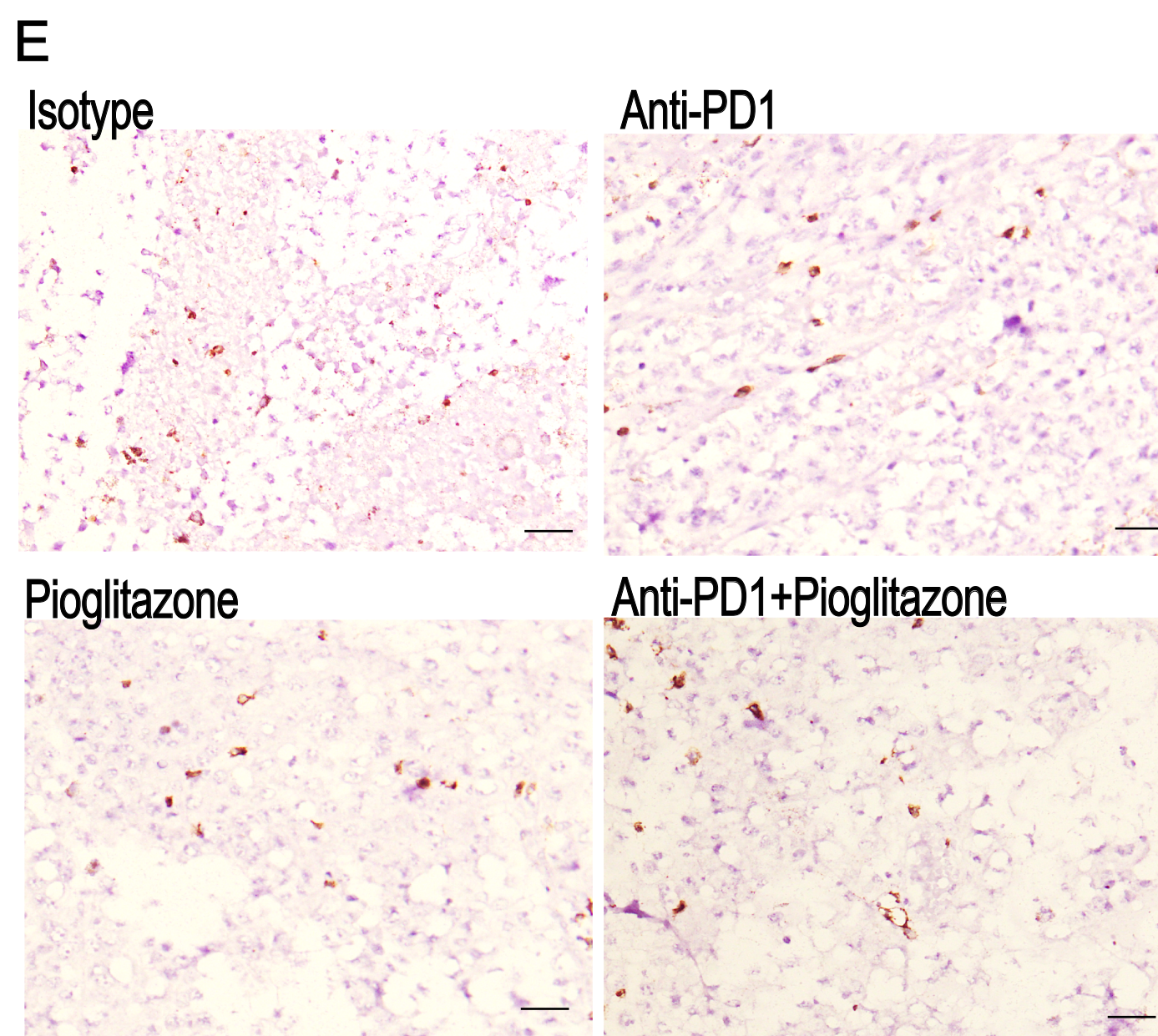

B16F10 tumor in C57BL/6 mice

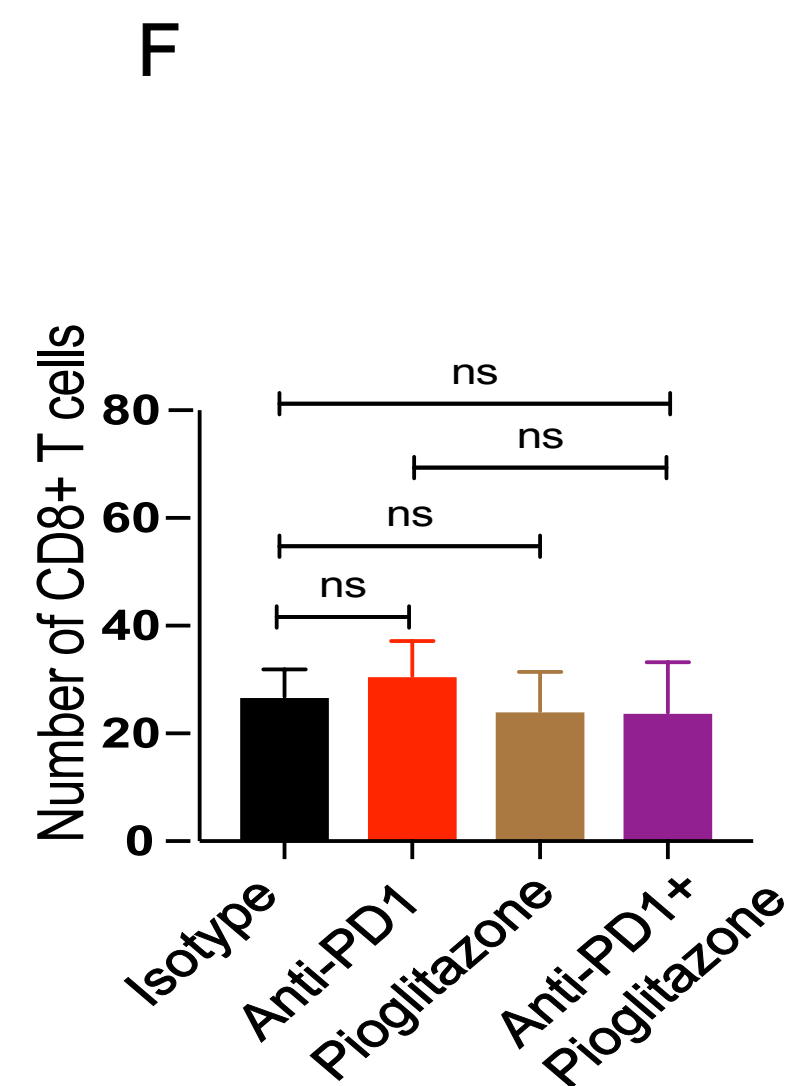

Supplement: Supplementary Materials — Fig. S1: acarbose inhibits melanoma tumor growth and enhances tumor immune responses to anti-PD1. Figure S2: sitagliptin has no effects on melanoma tumor growth and tumor immune responses to anti-PD1. Figure S3: metformin has no effects on melanoma tumor growth and tumor immune responses to anti-PD1. Figure S4: glimepiride enhances melanoma tumor immune responses to anti-PD1. Figure S5: pioglitazone inhibits melanoma tumor growth, but anti-PD1 weakens tumor inhibition of pioglitazone. Figure S6: insulin has no effects on melanoma tumor growth and tumor immune responses to anti-PD1. Figure S7: compare the effect of the six antidiabetic drugs on MC38 tumor inhibition. Figure S8: compare the effect of the six antidiabetic drugs on CT26 tumor inhibition. Figure S9: compare the effect of the six antidiabetic drugs on B16F10 tumor inhibition. Figure S10: the expression of IGF1R, IGF2R, and PPARG was negatively correlated with the number of infiltrated CD8+ T cells in colorectal cancer. Figure S11: the inhibitory effect of each antidiabetic drugs on CT26 cell proliferation. Figure S12: the effect of acarbose and insulin on anti-PD1 tumor inhibition was not related to blood glucose. Figure S13: the mice weight of each group in the day of MC38 tumor harvested. Table.S1: the weight of tumor after different Intervention [file 2651790.f1.zip › Figure S5.pdf]

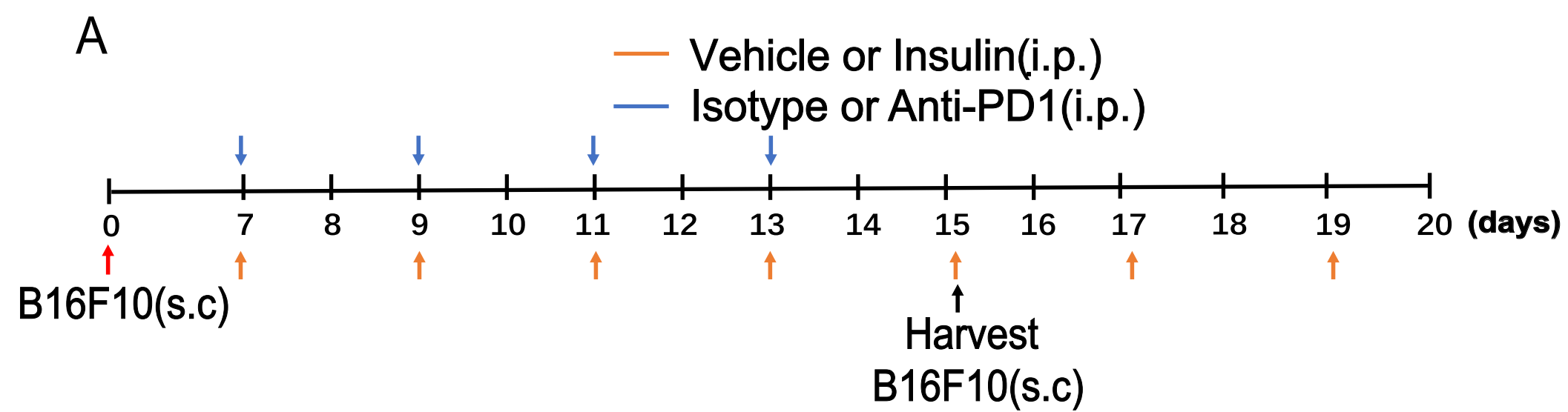

## B16F10 tumor in C57BL/6 mice

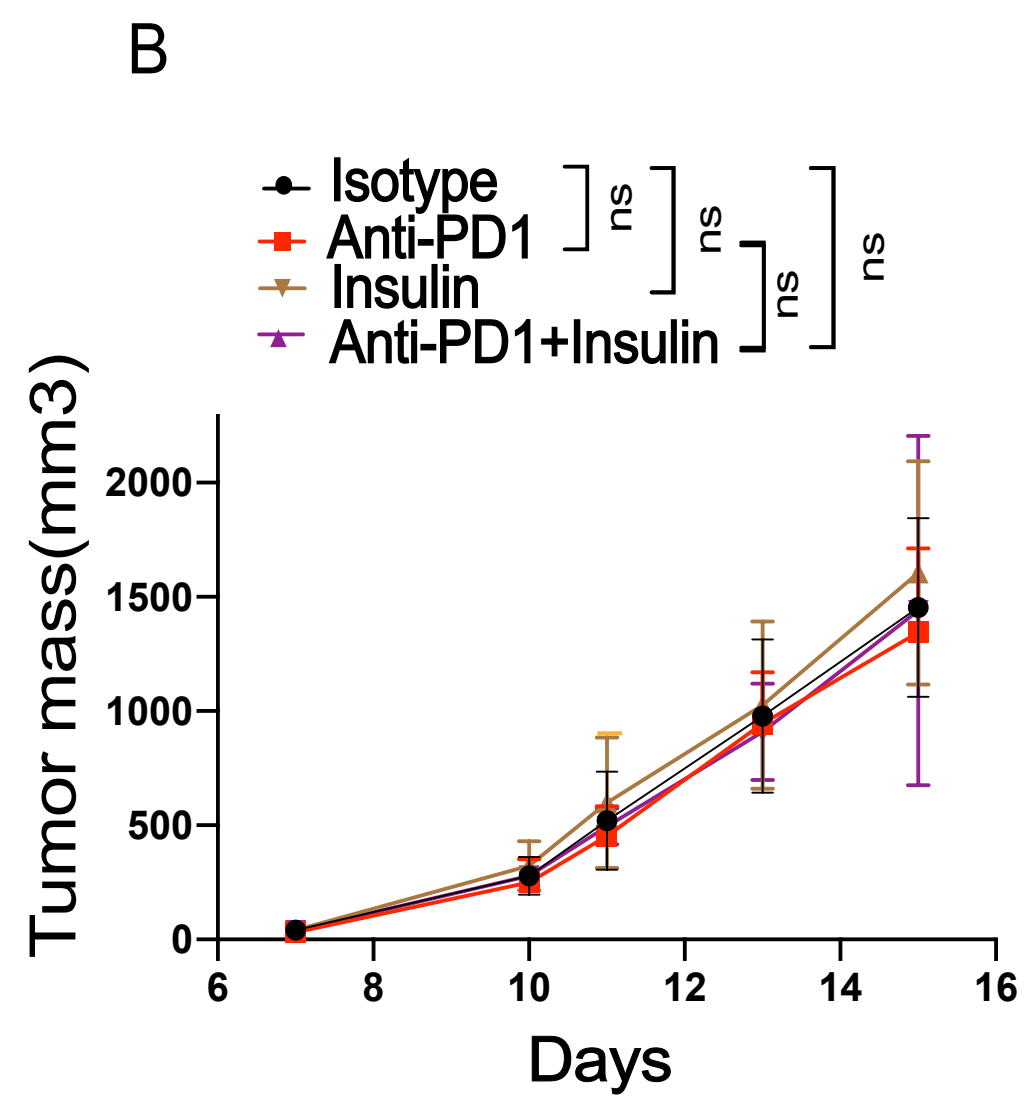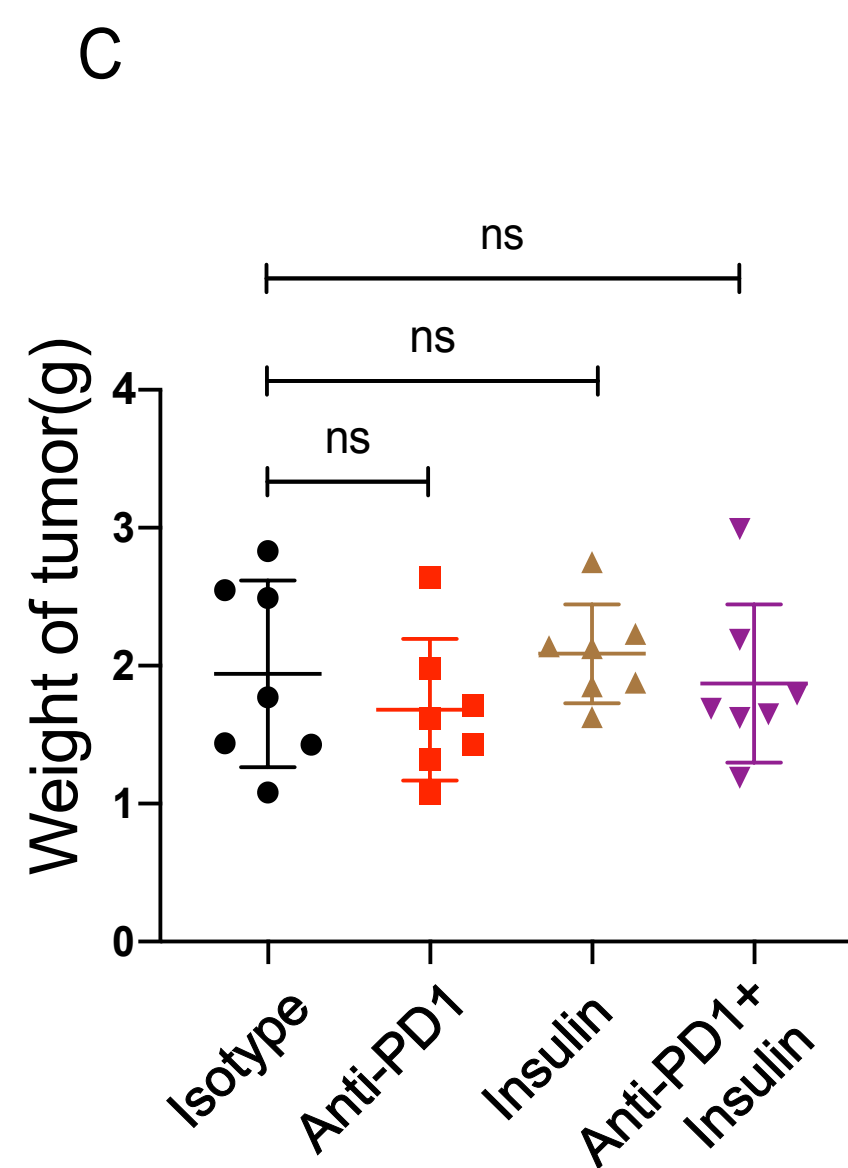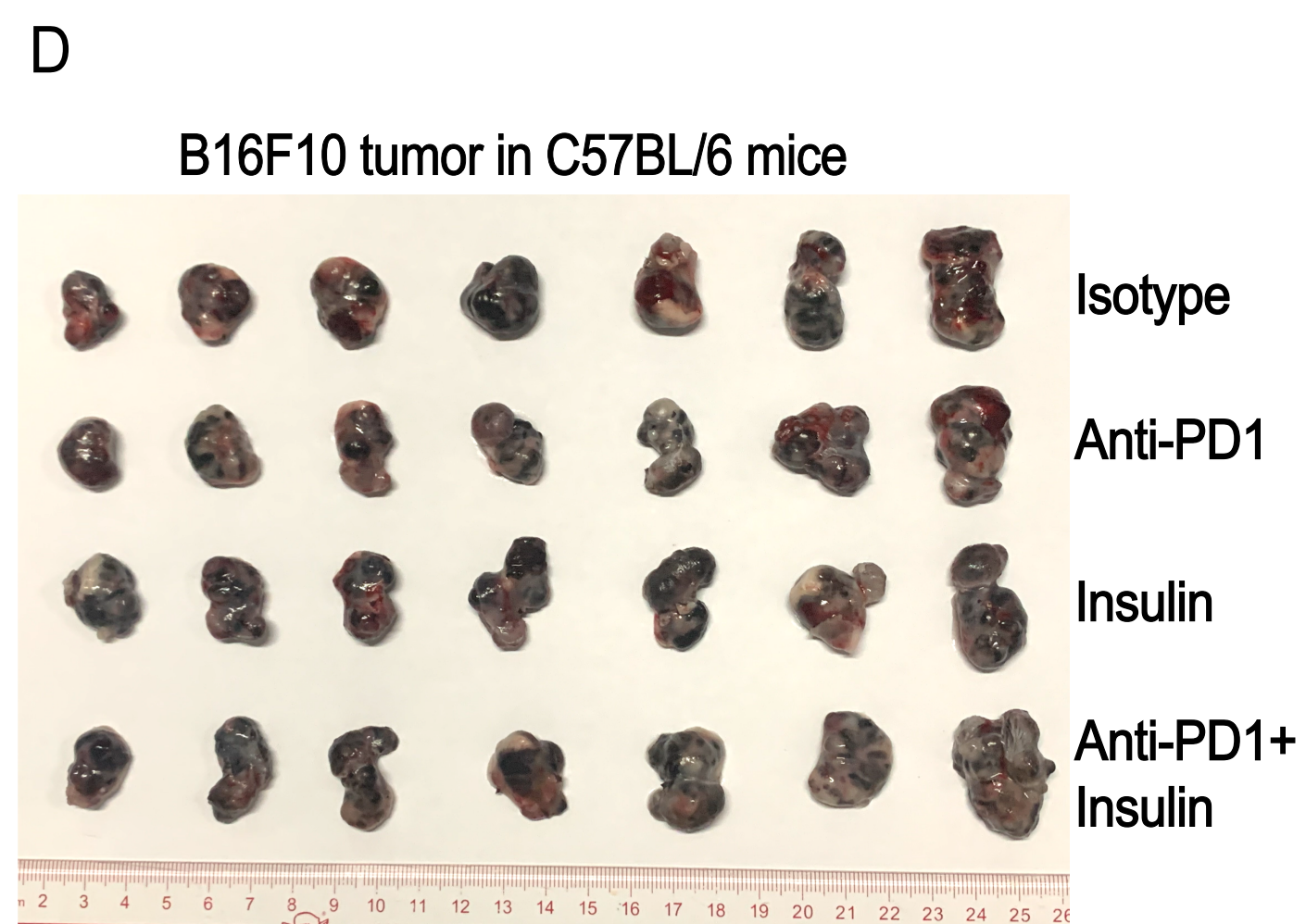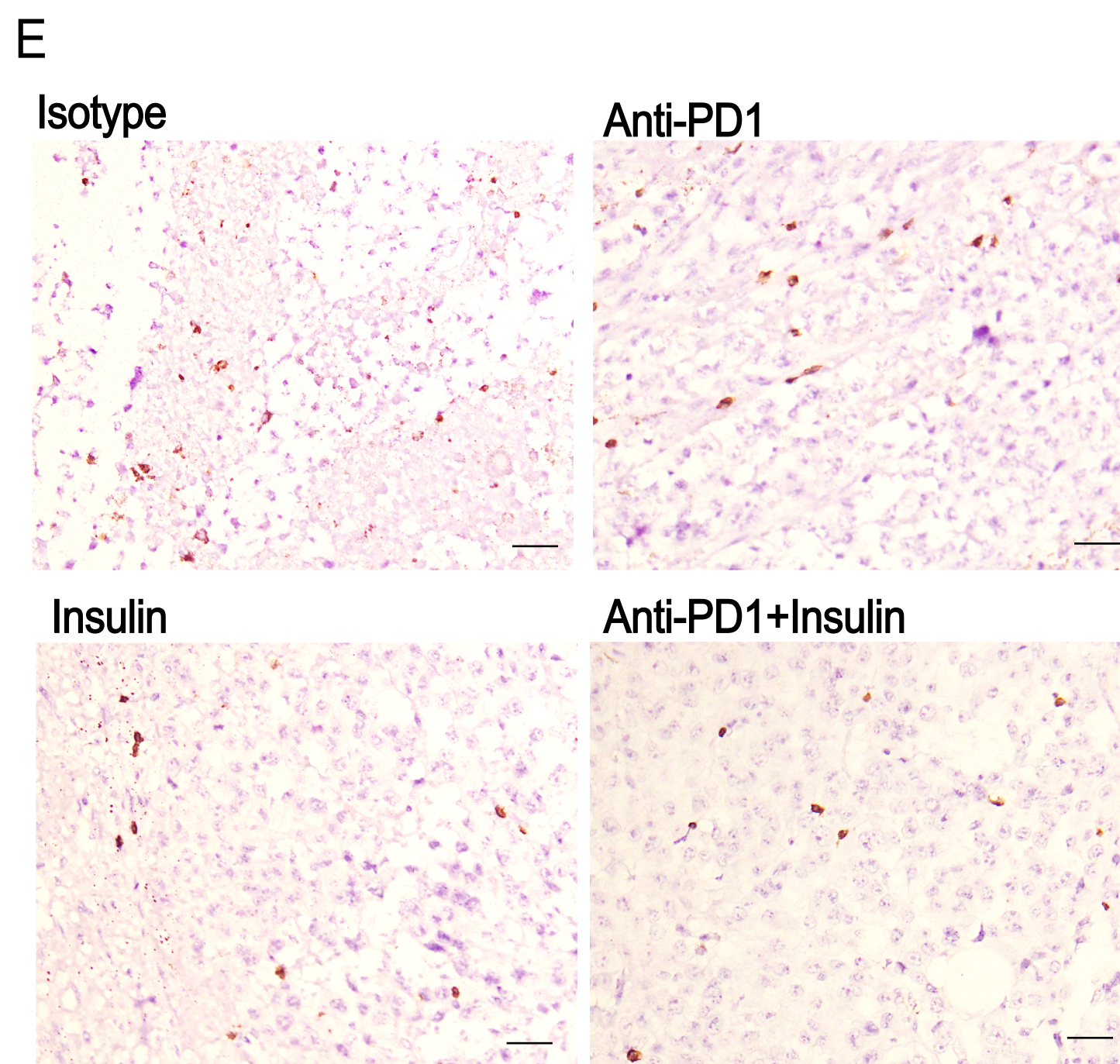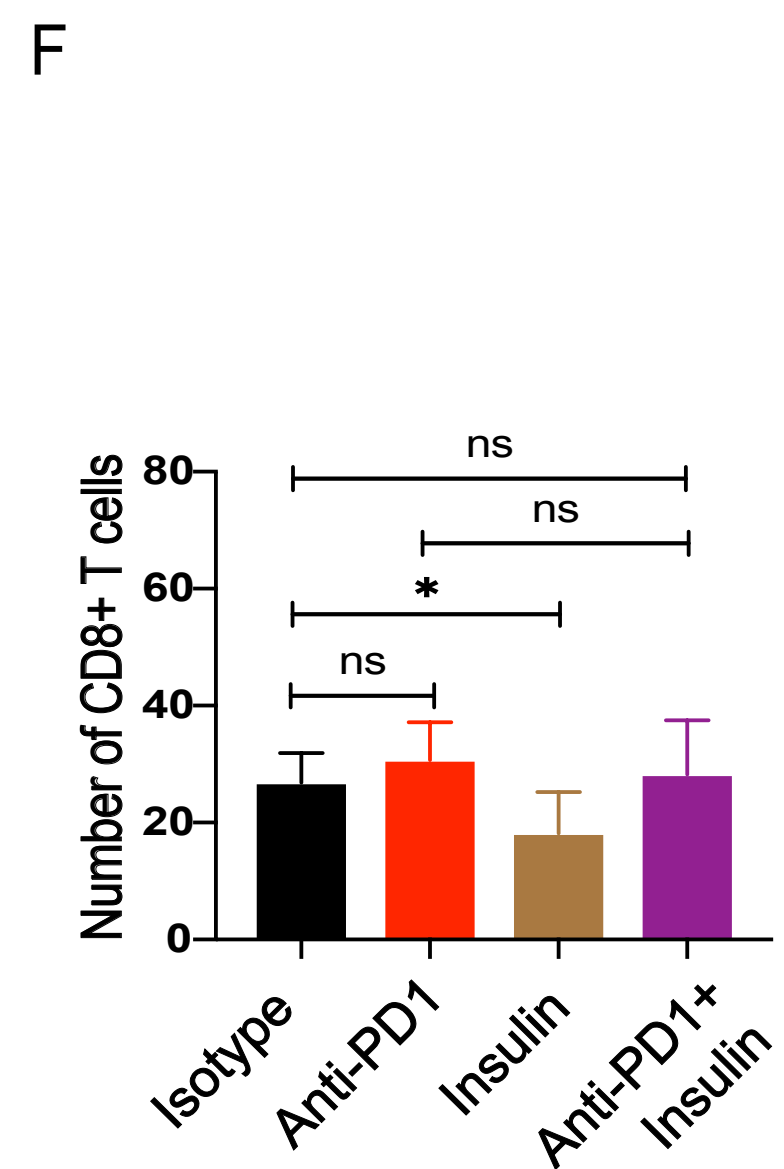

B16F10 tumor in C57BL/6 mice

Supplement: Supplementary Materials — Fig. S1: acarbose inhibits melanoma tumor growth and enhances tumor immune responses to anti-PD1. Figure S2: sitagliptin has no effects on melanoma tumor growth and tumor immune responses to anti-PD1. Figure S3: metformin has no effects on melanoma tumor growth and tumor immune responses to anti-PD1. Figure S4: glimepiride enhances melanoma tumor immune responses to anti-PD1. Figure S5: pioglitazone inhibits melanoma tumor growth, but anti-PD1 weakens tumor inhibition of pioglitazone. Figure S6: insulin has no effects on melanoma tumor growth and tumor immune responses to anti-PD1. Figure S7: compare the effect of the six antidiabetic drugs on MC38 tumor inhibition. Figure S8: compare the effect of the six antidiabetic drugs on CT26 tumor inhibition. Figure S9: compare the effect of the six antidiabetic drugs on B16F10 tumor inhibition. Figure S10: the expression of IGF1R, IGF2R, and PPARG was negatively correlated with the number of infiltrated CD8+ T cells in colorectal cancer. Figure S11: the inhibitory effect of each antidiabetic drugs on CT26 cell proliferation. Figure S12: the effect of acarbose and insulin on anti-PD1 tumor inhibition was not related to blood glucose. Figure S13: the mice weight of each group in the day of MC38 tumor harvested. Table.S1: the weight of tumor after different Intervention [file 2651790.f1.zip › Figure S6.pdf]

A

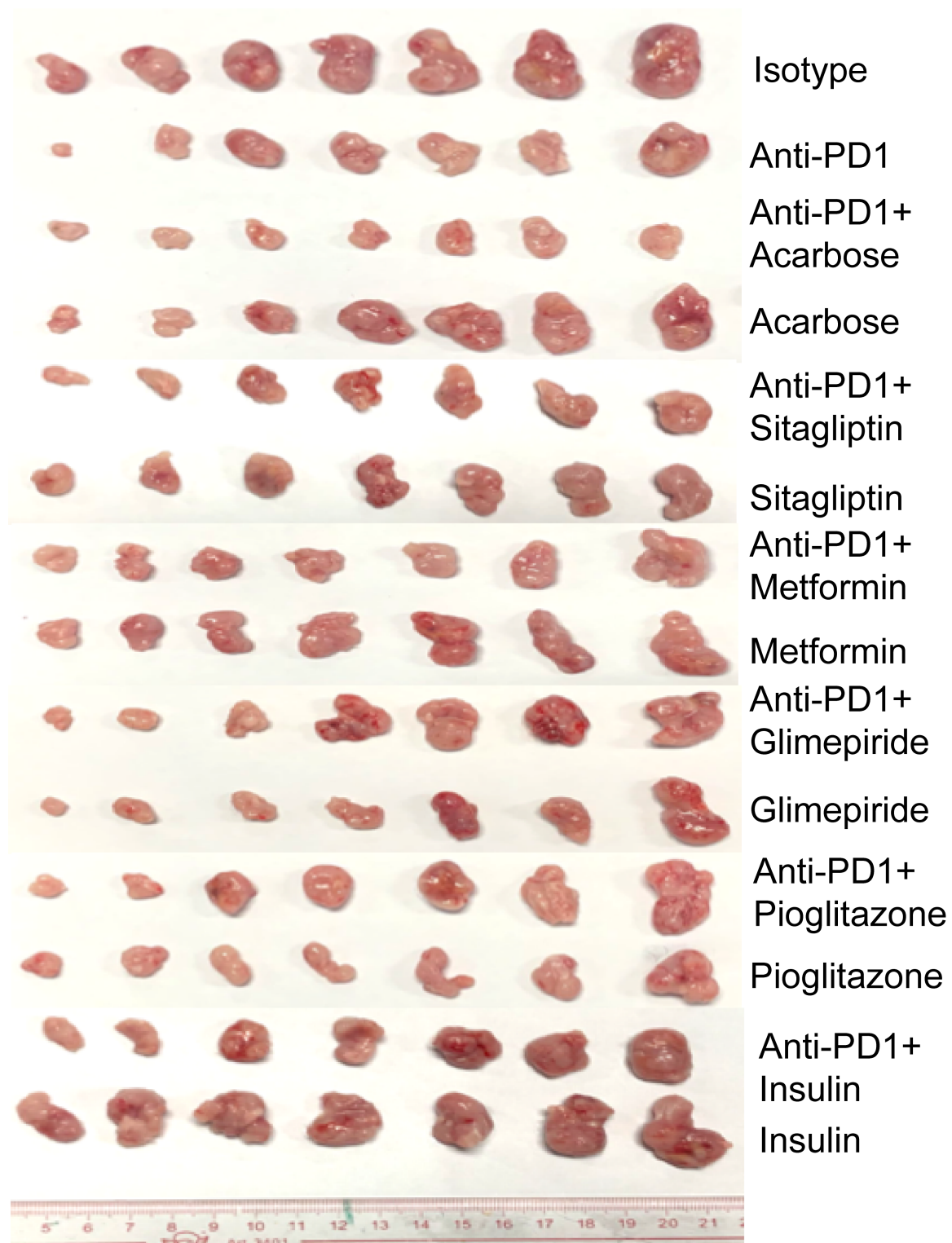

B

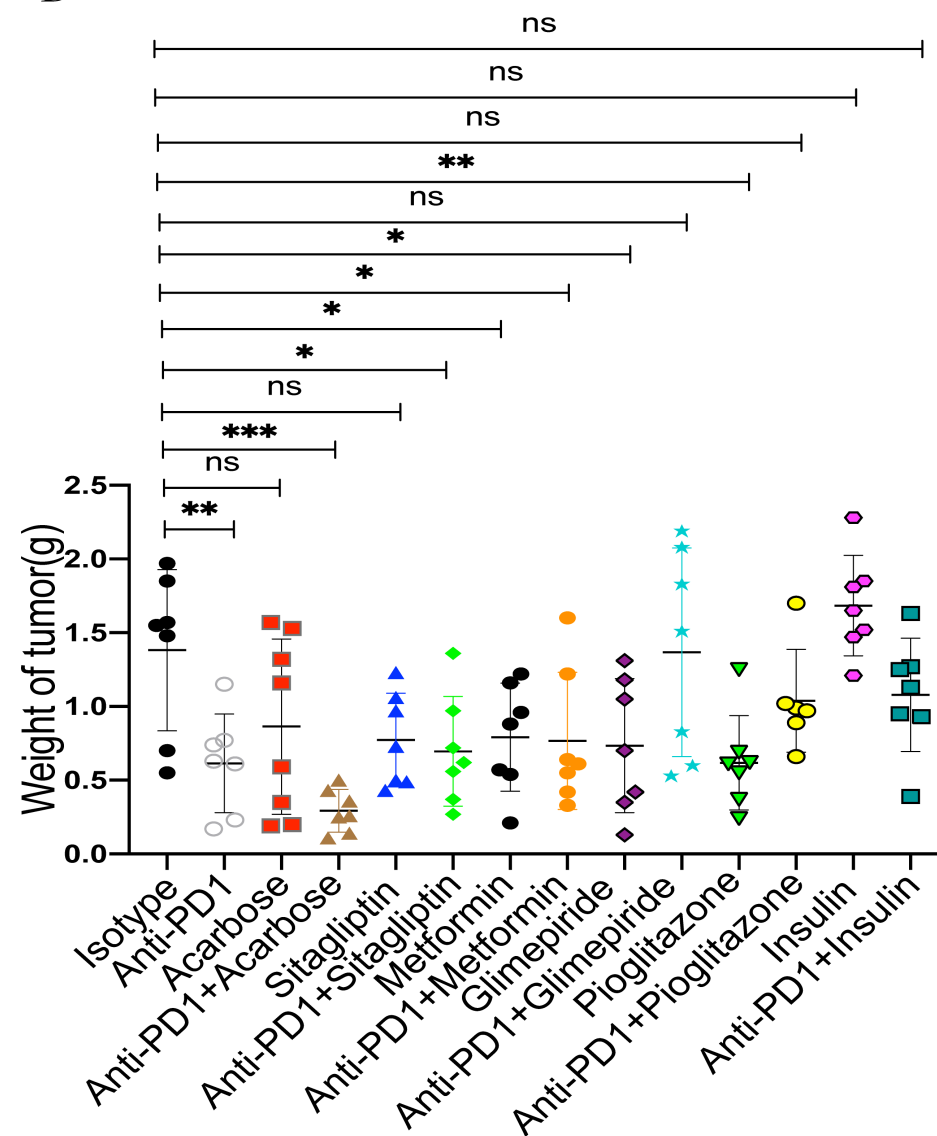

Supplement: Supplementary Materials — Fig. S1: acarbose inhibits melanoma tumor growth and enhances tumor immune responses to anti-PD1. Figure S2: sitagliptin has no effects on melanoma tumor growth and tumor immune responses to anti-PD1. Figure S3: metformin has no effects on melanoma tumor growth and tumor immune responses to anti-PD1. Figure S4: glimepiride enhances melanoma tumor immune responses to anti-PD1. Figure S5: pioglitazone inhibits melanoma tumor growth, but anti-PD1 weakens tumor inhibition of pioglitazone. Figure S6: insulin has no effects on melanoma tumor growth and tumor immune responses to anti-PD1. Figure S7: compare the effect of the six antidiabetic drugs on MC38 tumor inhibition. Figure S8: compare the effect of the six antidiabetic drugs on CT26 tumor inhibition. Figure S9: compare the effect of the six antidiabetic drugs on B16F10 tumor inhibition. Figure S10: the expression of IGF1R, IGF2R, and PPARG was negatively correlated with the number of infiltrated CD8+ T cells in colorectal cancer. Figure S11: the inhibitory effect of each antidiabetic drugs on CT26 cell proliferation. Figure S12: the effect of acarbose and insulin on anti-PD1 tumor inhibition was not related to blood glucose. Figure S13: the mice weight of each group in the day of MC38 tumor harvested. Table.S1: the weight of tumor after different Intervention [file 2651790.f1.zip › Figure S8..pdf]
